# Prioritization of causal genes from genome-wide association studies by Bayesian data integration across loci

**DOI:** 10.1371/journal.pcbi.1012725

**Published:** 2025-01-07

**Authors:** Zeinab Mousavi, Marios Arvanitis, ThuyVy Duong, Jennifer A. Brody, Alexis Battle, Nona Sotoodehnia, Ali Shojaie, Dan E. Arking, Joel S. Bader

**Affiliations:** 1 Department of Biomedical Engineering, Johns Hopkins University, Baltimore, Maryland, United States of America; 2 Institute for Computational Medicine, Johns Hopkins University, Baltimore, Maryland, United States of America; 3 Department of Genetic Medicine, Johns Hopkins University School of Medicine, Baltimore, Maryland, United States of America; 4 Cardiovascular Health Research Unit, Department of Medicine, University of Washington, Seattle, Washington, United States of America; 5 Malone Center for Engineering in Healthcare, Johns Hopkins University, Baltimore, Maryland, United States of America; 6 Department of Biostatistics, University of Washington, Seattle, Washington, United States of America; Thomas Jefferson University, UNITED STATES OF AMERICA

## Abstract

Motivation: Genome-wide association studies (GWAS) have identified genetic variants, usually single-nucleotide polymorphisms (SNPs), associated with human traits, including disease and disease risk. These variants (or causal variants in linkage disequilibrium with them) usually affect the regulation or function of a nearby gene. A GWAS locus can span many genes, however, and prioritizing which gene or genes in a locus are most likely to be causal remains a challenge. Better prioritization and prediction of causal genes could reveal disease mechanisms and suggest interventions.

Results: We describe a new Bayesian method, termed SigNet for significance networks, that combines information both within and across loci to identify the most likely causal gene at each locus. The SigNet method builds on existing methods that focus on individual loci with evidence from gene distance and expression quantitative trait loci (eQTL) by sharing information across loci using protein-protein and gene regulatory interaction network data. In an application to cardiac electrophysiology with 226 GWAS loci, only 46 (20%) have within-locus evidence from Mendelian genes, protein-coding changes, or colocalization with eQTL signals. At the remaining 180 loci lacking functional information, SigNet selects 56 genes other than the minimum distance gene, equal to 31% of the information-poor loci and 25% of the GWAS loci overall. Assessment by pathway enrichment demonstrates improved performance by SigNet. Review of individual loci shows literature evidence for genes selected by SigNet, including *PMP22* as a novel causal gene candidate.

## Author summary

A motivation for the Human Genome Project was to identify the genetic causes of diseases. The first human genome was sequenced about twenty years ago, and since then genome-wide association studies (GWAS) have identified genetic variants that correlate with many human traits, including disease and disease risk. Usually the GWAS variant affects the regulation of the closest gene or the activity of its protein product, but about 20–30% of the time the effect involves another gene that can be hundreds of kilobases away. We describe a new method, SigNet, to identify which gene within each GWAS locus is most likely to be causal. SigNet uses within-locus information when it is available, for example ‘gold-standard’ genes from family-based Mendelian studies, and adds between-locus information from protein-protein and gene-regulatory interaction networks to create a holistic model of causal genes across all loci. In applications to cardiovascular disease, we show better ability to identify relevant pathways and suggest new candidate genes within several GWAS loci.

## Introduction

The Human Genome Project was motivated by the goal of discovering the genetic basis of disease. A milestone draft sequence of a human genome was achieved about twenty years ago. Genetic variation between individuals, primarily single-nucleotide polymorphisms (SNPs), then provided a substrate for identifying variants that correlate with human traits, including disease and disease risk. GWAS have used statistical analysis of large human cohorts to identify SNPs that are associated with individual phenotypes. Understanding which gene in a GWAS locus is responsible for the causal effect is a current challenge [1].

The challenge arises for two reasons. First, SNPs identified by a GWAS are statistical associations, not causal mechanisms. Linkage disequilibrium creates large blocks of correlated SNPs or haplotypes. Methods that predict functional consequences of variants are helpful [2], but often statistical measures are insufficient to distinguish which SNPs in a block are responsible for a causal effect. Second, even among causal variants, only a small fraction occur in protein-coding regions, and a small fraction of these cause amino acid changes that provide strong evidence implicating a particular gene. At the majority of loci, the causal variants occur in intergenic regions thought to regulate the expression of nearby genes, but without direct evidence from GWAS of which gene’s regulation is affected.

Connecting SNPs to causal mechanisms is important when considering approaches to prevent or treat disease. A search for therapies often requires identifying a gene or protein target whose activity can be perturbed by a small molecule or biologic, or in more recent approaches by gene editing. The gene whose activity is affected directly by a GWAS SNP could be such a target, and could identify a downstream pathway with additional targets.

A default approach is to select the gene closest to a GWAS SNP as most likely to be causal. Many methods incorporate within-locus information to improve causal gene identification. A gene within the locus may already be known to be responsible for Mendelian forms of similar diseases or phenotypes, as recorded in databases such as OMIM [3]. Other methods use genetics of gene expression, often obtained from the GTEx database [4], to identify genes that are regulated by expression quantitative trait loci (eQTL) at the locus. One type of analysis, often termed colocalization, is performed at the level of individual variants, identifying GWAS SNPs that are also eQTL; methods include Coloc [5], eCaviar [6], eMagma [7], and ENLOC/fastENLOC [8, 9]. More recent studies have augmented expression cis-QTL with splicing cis-QTL for improved predictions [10].

A second type of analysis, an example being PrediXcan [11], builds a genetic predictor of gene expression to perform a transcriptome-wide association study, or TWAS. While colocalization and TWAS use similar or even identical data, the genes identified can be quite different [12, 13]. Other methods use chromatin state as within-locus evidence [14, 15]. Many QTL depend on cell type and developmental stage, however, and the cell type relevant to a particular disease may not be clear or may not be represented in GTEx or related databases. Furthermore, even if the relevant cell types are known and data are available, a locus may remain information poor. Our goal is to use evidence from information-rich loci to guide causal gene selection at information-poor loci.

We highlight examples of previous efforts in this and related areas. An early effort by Marcotte and coworkers used networks of functional associations (physical interactions augmented with coexpression and other evidence) to boost GWAS signals for genes near SNPs that may not have reached statistical significance. Bayes scores from GWAS were propagated to generate scores for all genes in the genome [16]. This problem is distinct from our focus on identifying the most likely gene at each locus given ample cohort sizes for statistical significance.

Many integrative analysis methods identify the most relevant genes within the set of genes with minimum distance to a GWAS SNP, generally equivalent to the mapped gene reported in the GWAS Catalog [17], then use physical interactions to identify relevant genes within this minimum distance set or interacting with them. A study by Ratnakumar et al. used such an approach to suggest core genes for disease phenotypes, including both genes identified through GWAS and genes associated through protein interactions [18]. In another study, genes not identified as causal by GWAS but linked through protein interactions have been suggested as drug targets [19].

Other methods have a similar goal to ours but with focus on a single type of data rather than on principled integration of multiple types of evidence. Roth and coworkers used functional association networks, including curated annotations, to select causal genes at GWAS loci [20]. They discussed the possibility of information leakage through annotations; we instead restrict our data sources to experimental data rather than annotated pathway membership. A study of GWAS in maize was similarly focused on functional associations, in this case from co-expression, rather than integration across data types [21]. It is also noteworthy in demonstrating that the problem of selecting causal genes is not specific to human but is more general across organisms.

Finally, methods to combine GWAS and non-GWAS data continue to use a simple set intersection approach, reporting genes highly ranked by both methods, rather than developing more systematic approaches. A study by Ferrari et al. used Mendelian genes as seeds in an interaction network to define a disease network that was intersected with candidate genes at GWAS loci [22]. A recent report by Finucane and coworkers calculated a polygenic priority score (PoPS) as a regression fit for gene-based GWAS scores using non-GWAS features, primarily gene expression data and protein interaction indicator functions [23]. Genes with locally maximal regression scores were then intersected with genes with GWAS significance.

**Fig 1:**
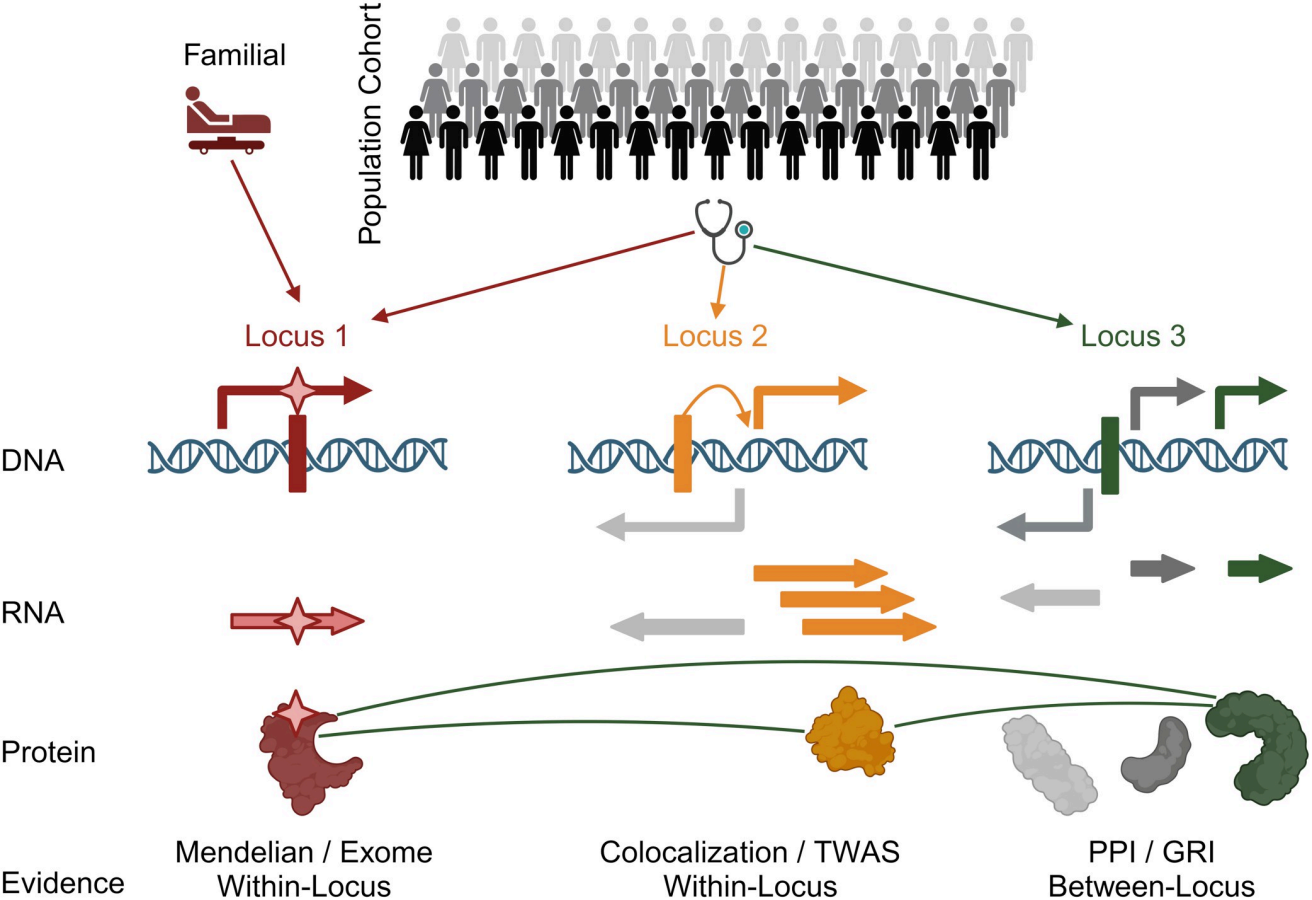
SigNet overview. Population cohorts (top) are genotyped and phenotyped in a genome-wide association study (GWAS). The study identifies genetic variants, usually single-nucleotide polymorphisms (SNPs, indicated by vertical bars overlayed on double-stranded DNA), that are associated with the phenotype at genome-wide significance. These SNPs occur throughout the genome, and each SNP defines a genomic region, or locus, that likely contains a gene with a causal relationship with the phenotype. Each locus may contain several genes (arrows above and below the double helix indicate genes on the positive and negative strand), and three loci are depicted. The SigNet method integrates within-locus and between-locus information from DNA-based, RNA-based, and protein-based evidence to select the most likely causal gene at each locus. **Locus 1 (red):** a SNP in a protein-coding region may change the amino acid sequence of the encoded protein, indicated by the star overlaying the gene symbol and protein. Similarly, a gene in the region may be known to cause a Mendelian disease related to the GWAS phenotype, indicated as a familial case. At this locus, the red gene is selected as most likely. **Locus 2 (orange):** a SNP may affect the transcriptional regulation of a nearby gene, indicated by the orange arrow from the SNP to the gene transcription start site. The corresponding mRNA transcript may have altered abundance, indicated by the multiple transcripts. These SNPs are expression quantitative trait loci (eQTL), and colocalization of a GWAS association with an eQTL association provides evidence for the most likely causal gene. Methods such as transcriptome-wide association studies (TWAS) provide a similar type of evidence. **Locus 3 (green):** Many loci are information-poor, with no within-locus evidence and a default approach of selecting the gene closest to the SNP. The SigNet method adds between-locus information using a probability model for the network formed by protein-protein interactions and gene-regulatory interaction of the genes selected at each locus. The green gene product interacts with proteins encoded by genes selected at the other loci, and its causal likelihood is calculated to be higher than the other genes in the locus, including the gene closest to the GWAS SNP.

These prior studies highlight the continuing need to explore methods that provide a principled integration of GWAS results with other biological data to identify the most likely causal genes at GWAS loci. The new method we describe, Signet for significance networks, focuses on loci that reach genome-wide significance and uses machine learning to predict the most likely causal gene within each locus. In addition to within-locus information such as distance from the significant SNP, protein-coding variation, and colocalization with eQTL, Signet uses between-locus interaction data: genes selected based on strong functional information at some loci can influence the genes selected at other loci (Fig 1). The between-locus data sets we consider are protein-protein interactions between gene products and gene-regulatory interactions between transcription factors and target genes. These types of interactions are enriched among genes and proteins that participate in related biological processes or contribute to similar phenotypes [24–26]. We draw information across all loci by favoring genes that form regulatory networks or signal transduction pathways with other selected genes. Stochastic block models for interaction enrichment, which we have used previously to discover hierarchical structure in biological networks [27, 28], here provide a principled framework to convert high-throughput data and biological intuition into a computable and interpretable probability distribution for identifying the most likely causal genes at GWAS loci.

We have applied this method to cardiac electrophysiology, chosen based on the availability of recent large GWAS and scientific expertise to evaluate prioritized genes. We describe how our predictions differ from closest-gene predictions and compare them with other available methods. Finally, we suggest possible extensions that incorporate additional types of evidence and that permit multiple causal genes at a single locus.

## Materials and methods

### Genome-wide association data

Genome-wide association data sets were downloaded from the GWAS Catalog [17]. Based on our ongoing participation in GWAS for cardiac electrophysiology [29], we selected electrocardiogram (EKG) parameters PR interval [30], QT interval [31], QRS interval [32], and JT interval [33] for analysis. We also included heart rate (HR) [34], which is used to correct the EKG parameters. Associations at the conventionally accepted p-value of 5 × 10^−8^ for genome-wide significance were retained. We mapped rsIDs to human genome assembly GRCh38.p13 released on July 2014 on Ensembl. Transcription start sites (TSSs) of each annotated gene were also obtained from the GRCh38.p13 assembly.

We created candidate regions, termed ‘GWAS loci’ throughout, by mapping each SNP to all protein-coding genes whose TSS was within a maximum distance *D* from the SNP. If no gene was found within the flanking region, we extended the distance to include the closest gene. Next, we aggregated SNP regions sharing at least one gene into a candidate locus. We used a distance *D* = 250 kb for our analysis and ascertained that results were not overly sensitive to smaller and larger values of *D* (see Results).

### Within-locus functional evidence: Mendelian genes, protein-coding variants, and colocalization

Exome chip data sets were collected for QT and JT intervals [35]. The protein-coding variants reported to reach genome-wide significance were retained.

Colocalization between a GWAS locus and cis-expression QTL (cis-eQTL), from the Coloc method [5], allows us to infer shared causal signals between the GWAS trait and the expression of nearby genes for each locus. For each genome-wide significant locus in at least one of the GWAS mentioned above, Coloc was applied for all genes for which the sentinel GWAS SNP was a genome-wide eQTL in heart tissues, specifically the Atrial Appendage and Left Ventricle, in GTEx v8 [4]. The Coloc with the Approximate Bayes Factor method was then run jointly between the GWAS and each eQTL gene-tissue pair including all SNPs in the region ±500 kb from the sentinel GWAS SNP that were also within 1 Mb of the gene TSS in that tissue. The Coloc parameters were set to recommended values of *p*_1_ = 10^−4^, *p*_2_ = 10^−4^, and *p*_12_ = 10^−6^. Despite the larger flank distance of 1 Mb for Coloc versus 250 kb for building GWAS loci, all genes identified by Coloc were within the GWAS loci.

Mendelian genes were gathered from OMIM [3] for these cardiovascular phenotypes: Brugada syndrome (BRGDA), Catecholaminergic polymorphic ventricular tachycardia (CPVT), Jervell and Lange-Nielsen syndrome (JLN), Long QT, Short QT, Sick sinus syndrome (SSS), and Wolff-Parkinson-White (WPW). While many Mendelian genes occurred within previously defined GWAS loci, several Mendelian genes occurred outside GWAS loci. These genes were retained by defining new single-gene loci. The GWAS loci together with the singleton Mendelian loci are termed the ‘total loci’.

### Cross-locus interaction evidence: Protein-protein and gene-regulatory interactions

We collected 236,584 Protein-Protein Interactions (PPI) from the Integrated Interactions Database [36], where we used the experimental and orthologous interactions in the 2021-05 version of the dataset. Interactions were treated as unweighted, undirected edges in a graph whose vertices represented proteins. Gene symbols and protein identifiers were mapped to each other using UniProt release 2015_06 [37]. Gene regulatory interactions (GRI) between transcription factor (TF) proteins and their targets were obtained from the TRRUST v2 database [38], with 8444 regulatory interactions for 800 TFs in humans. Interactions were treated as unweighted, directed edges in a graph whose vertices represented transcription factor sources and targets.

### Bayesian model selection

The probability distributions we consider couple together the observed data at individual loci with protein-protein and gene-regulatory interactions that cross between loci. We introduce notation **L** to represent the set of GWAS loci, with cardinality *L* = |**L**|, and similarly **M** to represent the set of singleton Mendelian genes falling outside GWAS loci, with cardinality *M* = |**M**|. The number of genes within locus *l* is *N*_*l*_; the number of genes in GWAS loci is *G*,

$$\begin{array}{c} G=\sum_{l\in L}N_{l}; \end{array}$$ (1)

and the total number of genes is *G* + *M*. Mendelian genes that are within GWAS loci are counted in *G*, not in *M*. An allowed configuration selects a single causal gene at each locus, termed the active gene. At a singleton Mendelian locus, the singleton Mendelian gene is always the active gene.

The active gene at locus *l* is denoted *a*_*l*_. The configuration defined by the set of active genes {*a*_*l*_} is denoted **a**. The set of active genes **a** defines a complementary set of inactive genes **b**_*l*_ for locus *l*. The set of all inactive genes is termed **b**, with

$$\begin{array}{c} b=\cup_{l=1}^{L}b_{l}, \end{array}$$ (2)

the union of the sets of inactive genes across all the loci. With the constraint of a single active gene at each locus, the total number of active genes must always equal the total number of loci,

$$\begin{array}{c} |a|=L+M. \end{array}$$ (3)

The number of inactive genes is similarly fixed,

$$\begin{array}{c} |b|=G-L. \end{array}$$ (4)

We often omit **b** in notation because this set is defined by **a**.

Our goal is to identify the configuration of active genes **a** that is most likely given the observed data **D**. The total number of possible configurations is $\prod_{l=1}^{L}N_{l}$, which grows combinatorially large with the number of GWAS loci. These configurations are assumed to follow a probability distribution, denoted Pr(**a**|**D**). For non-trivial probability distributions, identifying the optimal configuration is an NP-hard problem.

To make progress, we use Bayes law,

$$\begin{array}{c} \text{Pr}(a|D)=\text{Pr}(D|a)\text{Pr}(a)/\text{Pr}(D). \end{array}$$ (5)

The total data set **D** comprises individual data features, **D** ≡ {**D**_*f*_}. These features, denoted *f*, include within-locus real-valued features (the distance of a gene to a GWAS SNP), within-locus binary features (indicators for genes with Mendelian, exome, or colocalization evidence), and between-locus features (presence or absence of protein-protein or gene-regulatory interactions between pairs of genes and gene products). Formally,

$$\begin{array}{c} \text{Pr}(D|a)\equiv \text{Pr}(\{D_{f}\}|a). \end{array}$$ (6)

We make a simplifying naïve Bayes assumption of data independence,

$$\begin{array}{c} \text{Pr}\left(\left\{D_{f}\right\}|a\right)\approx \prod_{f}\text{Pr}\left(D_{f}|a\right). \end{array}$$ (7)

We explore co-occurrence of features to support the rationale for the naïve Bayes assumption (see Results).

We assumed a uniform prior, with each configuration having equal probability, $\text{Pr}(a)=\prod_{l=1}^{L}1/N_{l}$. The probability of the data Pr(**D**) is independent of the configuration **a**. The score *S*(**a**) of configuration **a** is defined as the log-likelihood ignoring these constant factors,

$$\begin{array}{c} S\left(a\right)\equiv \text{ln}\;\prod_{f}\text{Pr}\left(D_{f}|a\right)=\sum_{f}\text{ln}\;\text{Pr}\left(D_{f}|a\right)=\sum_{f}S_{f}\left(a\right). \end{array}$$ (8)

The probability distributions defining the scores have parameters that are shared across loci. These parameters are optimized using likelihood maximization, as described below.

### Distance score

The distance score *S*_Dist_ uses a parametric probability distribution to represent the observation that genes closer to a GWAS SNP are more likely to be causal. In the absence of other evidence, a weak effect is sufficient to bias selection of the closest gene. We therefore used an exponential decay for this probability distribution. Defining *x* as the distance from a gene’s transcription start site to the closest GWAS SNP, and *x* = 0 for singleton Mendelian genes, the distance score is

$$\begin{array}{c} S_{\text{Dist}}\left(x\right)\equiv \text{ln}\;[\frac{1}{2\gamma }\text{exp}\;\left(-|x|/\gamma \right)]. \end{array}$$ (9)

The multiplier 1/2*γ* is the standard factor ensuring normalization,

$$\begin{array}{c} \int_{-\infty }^{\infty }dx\;\text{exp}\;\left[S_{\text{Dist}}\left(x\right)\right]=1. \end{array}$$ (10)

The contribution of active genes to the distance score is therefore

$$\begin{array}{c} S_{\text{Dist}}\left(a\right)=\sum_{i\in a}-\text{ln}\;\left(2\gamma \right)-\left|x_{i}\right|/\gamma . \end{array}$$ (11)

The value of *γ* was updated at the end of each pass using maximum likelihood estimation, which requires (*d*/*dγ*)*S*_Dist_(**a**) = 0. The expression for the derivative yields the update

$$\begin{array}{c} \gamma =\frac{1}{L}\sum_{i\in a,i\notin M}\left|x_{i}\right|. \end{array}$$ (12)

Note that singleton Mendelian genes are excluded from contributing to the distance score update.

Let |**b**_*l*_| represent the number of inactive genes for locus *l*. The score for the inactive genes is represented by a uniform distribution,

$$\begin{array}{c} S_{\text{Dist}}\left(b\right)\equiv \frac{1}{|b_{l}|}. \end{array}$$ (13)

We consider a baseline where all the genes are inactive. Thus if we change a single gene from inactive to active, the score difference is *S*_Dist_(**a**) − *S*_Dist_(**b**). Since the score of inactive genes does not affect the relative gene scores of a locus, it is omitted from subsequent gene score calculation.

### Functional scores: Mendelian, exome, and colocalization evidence

The three categories of functional evidence (genes with evidence from Mendelian studies, exome variation, and colocalization) were treated individually with parameters *α*_*f*_ and *β*_*f*_, with *f* ∈ {Mendelian, Exome, Colocalization}:

$$\begin{array}{c} \begin{array}{cc} \text{Pr}(\text{has}\;\text{feature}|\text{not}\;\text{active})=\alpha & \text{Pr}(\text{lacks}\;\text{feature}|\text{not}\;\text{active})=1-\alpha \\ \text{Pr}(\text{has}\;\text{feature}|\text{active})=\beta & \text{Pr}(\text{lacks}\;\text{feature}|\text{active})=1-\beta . \end{array} \end{array}$$ (14)

For each locus, we introduce a fixed baseline score corresponding to all genes inactive,

$$\begin{array}{c} S_{0}=H\;\text{ln}\;\alpha +W\;\text{ln}\;\left(1-\alpha \right), \end{array}$$ (15)

where *H* is the number of genes having the feature and *W* is the number without the feature. If a gene having the feature is selected as active, the locus contributes a feature score ln(*β*/*α*); if a gene without the feature is selected as active, the locus contributes a feature score of ln[(1 − *β*)/(1 − *α*)]. We combined these into a single score equal to 0 if a gene without the feature is active and a score

$$\begin{array}{c} S_{f}=\text{ln}\;[\frac{\beta }{\alpha }]-\text{ln}\;[\frac{1-\beta }{1-\alpha }] \end{array}$$ (16)

if a gene with the feature is active.

At the end of each pass, the score *S*_*f*_ was updated for each categorical feature by maximizing the likelihood,

$$\begin{array}{c} \text{Pr}\left(\left\{n_{00},n_{01},n_{10},n_{11}\right\}|\alpha ,\beta \right)={\left(1-\alpha \right)}^{n_{00}}{\left(1-\beta \right)}^{n_{01}}\alpha^{n_{10}}\beta^{n_{11}}, \end{array}$$ (17)

where *n*_00_ is the number of inactive genes without the feature, *n*_01_ is the number of inactive genes with the feature, *n*_10_ is the number of active genes without the feature, and *n*_11_ is the number of active genes with the feature. We excluded the singleton Mendelian genes from these counts, giving a total count equal to the number of GWAS loci, *n*_00_ + *n*_01_ + *n*_10_ + *n*_11_ = *L*. As is often done, we added a pseudocount of 1 to each number to avoid undefined values. Parameters were updated by maximization with respect to *α* and *β*,

$$\begin{array}{c} S_{f}=\text{ln}\;[\frac{n_{11}+1}{n_{10}+1}]-\text{ln}\;[\frac{n_{01}+1}{n_{00}+1}]. \end{array}$$ (18)

### Degree-corrected network score

Many networks, including biological networks, such as protein-protein interaction (PPI) networks and gene-regulatory interaction (GRI) networks, have skewed degree distributions: some genes and proteins have many interactions, while others have few. We developed a degree-corrected network score to evaluate evidence based on a model in which interactions between active genes and proteins may be enriched relative to the null expectation.

For each type of network, denoted as net ∈ {PPI, GRI}, we counted the total number of interaction edges among the *L* + *M* active genes and their gene products, here including singleton Mendelian genes. Denoting the number of observed edges as *E*, we defined the log-likelihood ratio Λ_net_(*E*) for each network as

$$\begin{array}{c} \Lambda_{\text{net}}\left(E\right)=\text{ln}\;[\frac{\text{Pr}(E|\text{alt})}{\text{Pr}(E|\text{null})}], \end{array}$$ (19)

calculated separately for net = PPI and net = GRI. The alternative and null distributions have slightly different forms for the PPI and GRI networks because PPI interactions are generally modeled as undirected edges between interaction partners and GRI interactions are modeled as directed edges from transcription factor protein to target gene.

For the PPI network, edges are unweighted and undirected. Self-edges are ignored for two reasons. First, some technologies have difficulty identifying self-edges reliably. Second, methods that favor edge enrichment can create a bias in favor of selecting genes with self-edges.

The number of pairwise interactions among the *L* + *M* active proteins (here including the singleton Mendelian genes) is defined as *E*. Under the alternative hypothesis, the presence or absence of each edge is modeled as an independent, identically distributed binary random variable with success probability *θ*. The total number of pairs of active genes is denoted *T*, with

$$\begin{array}{c} T=(L+M)(L+M-1)/2. \end{array}$$ (20)

The probability of the observed count, conditioned on *θ*, is

$$\begin{array}{c} \text{Pr}\left(E|\theta \right)=\frac{T!}{E!(T-E)!}\theta^{E}{\left(1-\theta \right)}^{T-E}. \end{array}$$ (21)

The probability under the alternative hypothesis is obtained by integrating *θ* from 0 to 1,

$$\begin{array}{c} \text{Pr}\left(E|\text{alt}\right)=\int_{0}^{1}d\theta \;\text{Pr}\left(E|\theta \right)=\frac{1}{T+1}, \end{array}$$ (22)

a constant independent of the configuration.

The null hypothesis accounts for the vertex degrees by using the network defined by all *G* + *M* genes to define an effective *θ*_0_, or equivalently an effective *E*_0_ ≡ *Tθ*_0_. For the network defined by all genes *G* + *M* (here using all genes rather than just the active subset), let *E*_tot_ represent the total number of pairwise interactions and *d*_*i*_ the degree, or number of interaction partners, of protein *i*.

To build the null expectation, we use a standard degree-corrected interaction probability for proteins *i* and *j*. The *E*_tot_ edges have 2*E*_tot_ total endpoints. The probability that an edge with one endpoint at *i* has its other endpoint at *j* is therefore approximately *d*_*j*_/2*E*_tot_, with relative error on the order of *d*_*j*_/*E*_tot_. The probability that none of the *d*_*i*_ edges from *i* ends at *j* is approximately

$$\begin{array}{c} \text{Pr}\left(\text{no}\;\text{edge}\right)\approx {\left(1-\frac{d_{j}}{2E_{\text{tot}}}\right)}^{d_{i}}\approx \text{exp}\;(-\frac{d_{i}d_{j}}{2E_{\text{tot}}}). \end{array}$$ (23)

The probability of at least one edge between *i* and *j* is

$$\begin{array}{c} \text{Pr}\left(\text{edge}\right)\approx 1-\text{exp}\;(-\frac{d_{i}d_{j}}{2E_{\text{tot}}})\approx \frac{d_{i}d_{j}}{2E_{\text{tot}}}, \end{array}$$ (24)

with error terms on the order of (*d*_*i*_*d*_*j*_/2*E*_tot_)^2^. Therefore, provided that vertex degree products are smaller than the total number of edges, *d*_*i*_*d*_*j*_/2*E*_tot_ provides a degree-corrected edge probability. Note that these terms can be calculated once at the start of run and factorize conveniently. Therefore, after defining the GWAS and Mendelian loci with *G* + *M* total genes and *E*_tot_ interactions, we define

$$\begin{array}{c} \delta_{i}\equiv \frac{d_{i}}{\sqrt{2E_{\text{tot}}}} \end{array}$$ (25)

and store these values. Then, for the *L* active genes at GWAS loci and additional *M* singleton Mendelian genes, for convenience numbered *i* ∈ 1, 2, 3, …, *L* + *M*, the expected number of edges under the null is

$$\begin{array}{c} E_{0}=\sum_{i=1}^{L+M}\sum_{j=i+1}^{L+M}\delta_{i}\delta_{j}. \end{array}$$ (26)

We then use the standard limiting form of the binomial distribution as a Poisson distribution, $\text{Pr}(E|\text{null})=\text{Pr}(E|E_{0})=\left(E_{0}^{E}/E!\right)\text{exp}\;\left(-E_{0}\right)$.

The log-likelihood ratio for the PPI network is therefore

$$\begin{array}{c} \Lambda_{\text{PPI}}\left(E\right)=\text{ln}\;[\frac{E!\text{exp}\;(E_{0})}{\left(T+1\right)E_{0}^{E}}]=\text{ln}\;\Gamma \left(E+1\right)-E\;\text{ln}\;E_{0}+E_{0}-\text{ln}\;\left(T+1\right). \end{array}$$ (27)

The term Γ(*E* + 1) is the standard Γ function, with Γ(*k* + 1) = *k*!. While we do not use Stirling’s approximation, substituting the approximation that Γ(*E* + 1) ≈ *E* ln *E* − *E* yields the log-likelihood equivalent to a maximum-likelihood estimator,

$$\begin{array}{c} \Lambda_{\text{PPI}}^{\text{ML}}\left(E\right)\approx E\;\text{ln}\;\left(E/E_{0}\right)-\left(E-E_{0}\right)-\text{ln}\;\left(T+1\right). \end{array}$$ (28)

The minimum value of Λ_PPI_(*E*) occurs close to *E* = *E*_0_, and for $\Lambda_{\text{PPI}}^{\text{ML}}\left(E\right)$ occurs exactly at *E* = *E*_0_.

The network score Λ_PPI_(*E*) favors deviations of edge counts in both directions, enriched (the expected direction) and depleted. To avoid convergence to an edge-depleted state that may be a local optimum but is unlikely to be a global optimum, we define the network score *S*_PPI_(*E*) to favor edge enrichment over edge depletion:

$$\begin{array}{c} S_{\text{PPI}}\left(E\right)=\Lambda_{\text{PPI}}\left(E_{0}\right)+\text{sgn}\left(E-E_{0}\right)\left|\Lambda_{\text{PPI}}\left(E\right)-\Lambda_{\text{PPI}}\left(E_{0}\right)\right|, \end{array}$$ (29)

where sgn(*x*) is the sign function, +1 for positive *x*, −1 for negative *x*, and 0 for zero-valued *x*. The value of the log-likelihood ratio Λ_PPI_(*E*) at *E* = *E*_0_ from Eq 27 is used as a baseline, and the magnitude of the difference between the calculated value and the baseline value is added for edge enrichment (*E* > *E*_0_) and subtracted for edge depletion (*E* < *E*_0_).

The GRI network score uses a directed, unweighted, degree-corrected network model that yields results that are similar to the PPI network results, except that in-degree and out-degree are considered separately. Variables and parameters for the GRI network are distinguished from similar PPI notation by appending a ′ character. The total number of edges in the observed network is denoted *E*′, and the total number of possible edges, excluding self-edges as with the PPI network, is

$$\begin{array}{c} T^{\prime }=\left(G+M\right)\left(G+M-1\right). \end{array}$$ (30)

The log-likelihood under the alternative hypothesis is

$$\begin{array}{c} \text{Pr}\left(E^{\prime }|\text{alt}\right)=\int_{0}^{1}d\theta^{\prime }\;\text{Pr}\left(E^{\prime }|\theta^{\prime }\right)=\frac{1}{T^{\prime }+1}. \end{array}$$ (31)

Under the null hypothesis, the probability of an edge from vertex *j* to vertex *i*, denoted edge *ij*, is degree-corrected:

$$\begin{array}{c} \text{Pr}\left(\text{edge}\;ij\right)\approx \frac{d_{i,\text{in}}^{\prime }d_{j,\text{out}}^{\prime }}{E_{\text{tot}}^{\prime }}. \end{array}$$ (32)

These degrees are calculated from the entire network of all *G* + *M* genes at all loci, with $d_{i,\text{in}}^{\prime }$ as the in-degree of vertex *i*, $d_{j,\text{out}}^{\prime }$ as the out-degree of vertex *j*, and $E_{\text{tot}}^{\prime }$ as the total number of edges between all pairs of *G* + *M* genes at all *L* + *M* loci. After the loci are defined, degree-corrected parameters are calculated once at the beginning of the run:

$$\begin{array}{ccc} \delta_{i,\text{in}}^{\prime } & \equiv & \frac{d_{i,\text{in}}^{\prime }}{\sqrt{E_{\text{tot}}^{\prime }}} \end{array}$$ (33)

$$\begin{array}{ccc} \delta_{j,\text{out}}^{\prime } & \equiv & \frac{d_{j,\text{out}}^{\prime }}{\sqrt{E_{\text{tot}}^{\prime }}}. \end{array}$$ (34)

Then, following the same approach as for the PPI network, the score for the GRI network, *S*_GRI_(*E*′), is calculated as follows:

$$\begin{array}{ccc} E_{0}^{\prime } & = & \sum_{i=1}^{L+M}\sum_{j\neq i,j=1}^{L+M}\delta_{i,\text{in}}^{\prime }\delta_{j,\text{out}}^{\prime } \end{array}$$ (35)

$$\begin{array}{ccc} \Lambda_{\text{GRI}}\left(E^{\prime }\right) & = & \text{ln}\;\Gamma \left(E^{\prime }+1\right)-E^{\prime }\;\text{ln}\;E_{0}^{\prime }+E_{0}^{\prime }-\text{ln}\;\left(T^{\prime }+1\right) \end{array}$$ (36)

$$\begin{array}{ccc} S_{\text{GRI}}\left(E^{\prime }\right) & = & \Lambda_{\text{GRI}}\left(E_{0}^{\prime }\right)+\text{sgn}\left(E^{\prime }-E_{0}^{\prime }\right)\left|\Lambda_{\text{GRI}}\left(E^{\prime }\right)-\Lambda_{\text{GRI}}\left(E_{0}^{\prime }\right)\right|. \end{array}$$ (37)

As noted above, computational time is reduced by pre-calculating the *δ*_*i*_ values for the PPI network and the $\delta_{i,\text{in}}^{\prime }$ and $\delta_{j,\text{out}}^{\prime }$ values for the GRI networks. We considered three additional performance enhancements. First, rather than calculating *E*_0_ and $E_{0}^{\prime }$ by summing over all pairs, it is possible to sum first and then subtract off self-terms:

$$\begin{array}{ccc} E_{0} & = & \frac{1}{2}{\left[\sum_{i=1}^{L+M}\delta_{i}\right]}^{2}-\frac{1}{2}\sum_{i=1}^{L+M}\delta_{i}^{2} \end{array}$$ (38)

$$\begin{array}{ccc} E_{0}^{\prime } & = & [\sum_{i=1}^{L+M}\delta_{i,\text{in}}^{\prime }][\sum_{j=1}^{L+M}\delta_{j,\text{out}}^{\prime }]-\sum_{i=1}^{L+M}\delta_{i,\text{in}}^{\prime }\delta_{i,\text{out}}^{\prime }. \end{array}$$ (39)

Second, many configurations are revisited over multiple passes. The set of genes in a configuration can be used as a key to store the network score the first time a configuration is observed and then to retrieve the cached score if it is visited again. For further efficiency, the set of genes can be limited to genes that have non-zero vertex degree for the network type. Caching is particularly valuable for the sparse GRI network.

A third performance improvement, when visiting a particular locus, is to pre-calculate the observed and expected edge counts within all the other loci (again with caching), and then to only consider the new observed and expected edge counts from the locus being visited to the other *L* + *M* − 1 loci. We implemented the first and second improvements, which gave adequate performance.

### Initialization, sampling, and convergence

The active network is initially configured by selecting the most plausible causal gene at a locus as the selected gene. Mendelian genes are given the highest priority, followed by exome-chip and then colocalized genes. If a locus has no genes with functional evidence, the gene with the minimum distance is selected as the causal gene. Thus, our initial network configuration provides a baseline upon which SigNet improves. We then performed 100 independent runs, each pass traversing each locus in a random permuted order. For locus *l*, the active genes at all other loci are frozen, and we calculate the score *S*_*i*_ for each gene *i* of the *N*_*l*_ genes within locus *l* as the active gene *a*_*l*_,

$$\begin{array}{c} S_{i}=S_{\text{Distance}}+S_{\text{Mendelian}}+S_{\text{Exome}}+S_{\text{Colocalization}}+S_{\text{PPI}}+S_{\text{GRI}}. \end{array}$$ (40)

We define the weight *w*_*i*_ as the probability of gene *i* at locus *l* being active,

$$\begin{array}{c} w_{i}=\frac{\text{exp}\;(S_{i})}{\sum_{j=1}^{N_{l}}\text{exp}\;\left(S_{j}\right)}. \end{array}$$ (41)

We record the weights for each gene, {*w*_*i*_}, set the active gene at the locus to be the gene *m* with the maximum score, *a*_*l*_ = *m*, and proceeded to the next locus. If genes had tied scores, one is selected at random. At the end of each pass, probability distribution parameters are updated as described above. Runs continue until the set of active genes is unchanged. Since parameter values are updated based on active genes, this implies that the parameters are also unchanged. At the end of each of the 100 runs, we recorded the final, converged value of *w*_*i*_ for each gene and then computed the overall mean of *w*_*i*_ over the 100 runs. We also computed the frequency that each gene was selected as the active gene, again averaged over the 100 runs.

Note that the selection frequency can be different from the gene weight. If a gene has a weight above 0.5, it will always be selected, leading to a selection frequency of 1. An alternative to our greedy approach would be a Gibbs sampler, selecting the new configuration according to the gene weights. Gibbs samplers are appropriate when transitions between high-scoring configurations are frequent. Our greater concern is trapping in the region of one particular high-scoring configuration with rare transitions to other high-scoring configurations, and therefore we assessed convergence of the greedy sampler over multiple random restarts (see Results). Furthermore, we tested the performance of SigNet when the active network was initialized at random. For each run, the active genes were initialized by selecting one gene uniformly at random from each locus. We then performed 100 passes, each pass traversing each locus in a random permuted order (see Results).

### Gene selection by SigNet, SigNet+, and Mindist

We define the SigNet gene list as the single gene selected most often by SigNet over the 100 runs. Some loci contain multiple genes with functional evidence, including loci with multiple Mendelian genes. To avoid the limitation of selecting a single gene at these loci, we define the SigNet+ gene list as the union of the SigNet genes with the set of genes with any functional evidence from Mendelian studies, exome chips, or colocalization analysis. The MinDist method is a baseline approach of selecting the single gene within a locus with minimum distance from its transcription start site to the closest GWAS SNP.

We performed analysis over the full set of loci and over an information-poor set of loci, defined as loci lacking any genes with functional evidence. For the information-poor loci, SigNet and SigNet+ are necessarily equivalent.

### Implementation and performance

The SigNet method was implemented in Python with standard open-source libraries. The Graphviz library was used for graph drawing [39, 40] and the Fruchterman-Reingold force-directed placement algorithm was used for graph layout [41]. Computation time on a 2.9 GHz CPU, 32 GB memory, for the traits considered here was 15–20 sec per run, or about 30 min for the entire results. The SigNet software with documentation for installation and use is available under the BSD 2-Clause Simplified License at https://github.com/joelbaderlab/signet_v1 and from Zenodo under DOI 10.5281/zenodo.12774442 at https://zenodo.org/doi/10.5281/zenodo.12774442 [42].

## Results

### Cardiac electrophysiology GWAS loci

GWAS summary data sets were downloaded from the NHGRI-EBI GWAS Catalog [17] for the most recent studies of the following electrocardiogram (EKG) parameters: PR interval [30], QT interval [31], QRS interval [32], JT interval [33], and heart rate (HR) [34]. Studies except JT were ∼99% European ancestry cohorts. For JT, the ancestry was 63% European, 21% Hispanic/Latino, and 16% African American. Single-nucleotide polymorphisms (SNPs) were selected if the reported p-value was 5 × 10^−8^ or below (Table 1).

**Table 1:** Cardiovascular GWAS data.

Phenotype	GWAS		Genes	
	Cohort size	SNPs	Exome	Colocalized
JT	71,857	69	9	-
QRS	60,255	73	-	4
PR	92,340	44	-	11
QT	70,389	98	3	15
HR	134,251	86	-	12
**Total unique**		**345**	**12**	**38**

GWAS cohort size and number of SNPs for electrophysiology phenotypes. The number of genes with Exome and Colocalization evidence associated with each phenotype is also stated.

Exome-chip data from a recent study of 95,626 individuals from 23 cohorts identified 45 loci associated with ventricular repolarization, of which six were novel for QT, and four were novel for JT, implicating a total of 12 genes [35].

The phenotypes under study have 345 significant GWAS SNPs. With a flank distance of 250 kb, and merging loci with shared genes, the resulting network had 226 loci and 1165 genes. To assess robustness, we also constructed loci using 125 kb flanks and 500 kb flanks (Table 2). While the median locus width and number of genes per locus increase proportionally with the two-fold changes in flank size, the number of loci changes only by about 10%.

**Table 2:** Cardiovascular GWAS loci.

Flank distance	125 kb	250 kb	500 kb
Number of GWAS loci	240	226	210
Locus width, median	204	387	856
Genes, total	693	1167	2034
Genes per locus, median	2	3	7

Summary statistics for networks of GWAS loci constructed with flank distances of 125, 250, and 500 kb.

### Functional evidence

In addition to functional evidence from exome-chip data, functional evidence was also gathered from colocalization of GWAS signals with cis-eQTL using Coloc. Colocalization of GWAS signals with cis-eQTL in heart tissue identified 38 genes, with some identified in multiple phenotypes (Table 1). Mendelian genes for heritable forms of arrythmia were obtained from the Online Mendelian Inheritance in Man (OMIM) database [3]. Alleles were collapsed onto 31 single genes, with some genes linked to multiple phenotypes (Table 3). The 31 genes with Mendelian evidence, 12 genes with exome-chip evidence, and 38 genes with colocalization evidence had little overlap with each other and mapped to 75 unique genes with functional evidence. Of the 31 Mendelian genes, 12 were in GWAS loci. The 19 remaining genes were added as single-gene loci to yield 245 total loci.

**Table 3:** Cardiovascular Mendelian genes.

Phenotype	Genes
LongQT	18
BRGDA	9
ShortQT	6
CPVT	5
SSS	2
JLN	2
WPW	1
**Total unique**	**31**
Mendelian genes in GWAS	12
Singleton Mendelian genes	19

Mendelian genes linked to cardiovascular function were included in our analysis. The number of genes for each phenotype is shown. Abbreviations: BRGDA = Brugada syndrome, CPVT = Catecholaminergic polymorphic ventricular tachycardia, JLN = Jervell and Lange-Nielsen syndrome, SSS = Sick sinus syndrome, WPW = Wolff-Parkinson-White.

We analyzed the overlap of genes with functional evidence, restricted to the 1165 genes in GWAS loci, excluding the Mendelian genes without GWAS evidence (Table 4). While the overlap between Mendelian genes and exome genes is significant (*p* = 3.1 × 10^−6^), the number of genes with this shared evidence is small, only 4. The number of genes with other types of shared evidence is also small. The small number of genes with shared evidence supports the naïve Bayes approach to treat these different types of evidence as independent.

**Table 4:** Functional evidence.

**Category**	**Number of genes**		
Mendelian	12		
Exome	12		
Colocalized	38		
**Intersection**	**Genes**	**Count**	**P-value**
Mendelian–Exome	*KCNH2* *KCNQ1* *SCN10A* *SLC4A3*	4	3.1 × 10^−6^
Mendelian–Colocalized	*KCNJ5* *SCN10A*	2	0.056
Exome–Colocalized	*SCN10A*	1	0.33
Mendelian–Exome–Colocalized	*SCN10A*	1	0.0040

Genes are restricted to those within the 226 GWAS loci defined with 250 kb flanks and exclude the 19 additional singleton Mendelian genes. Significance tests for the number of genes in two categories are from Fisher exact tests and for three categories are from a binomial test.

### Robust convergence to a network of selected genes (SigNet and SigNet+)

**Fig 2:**
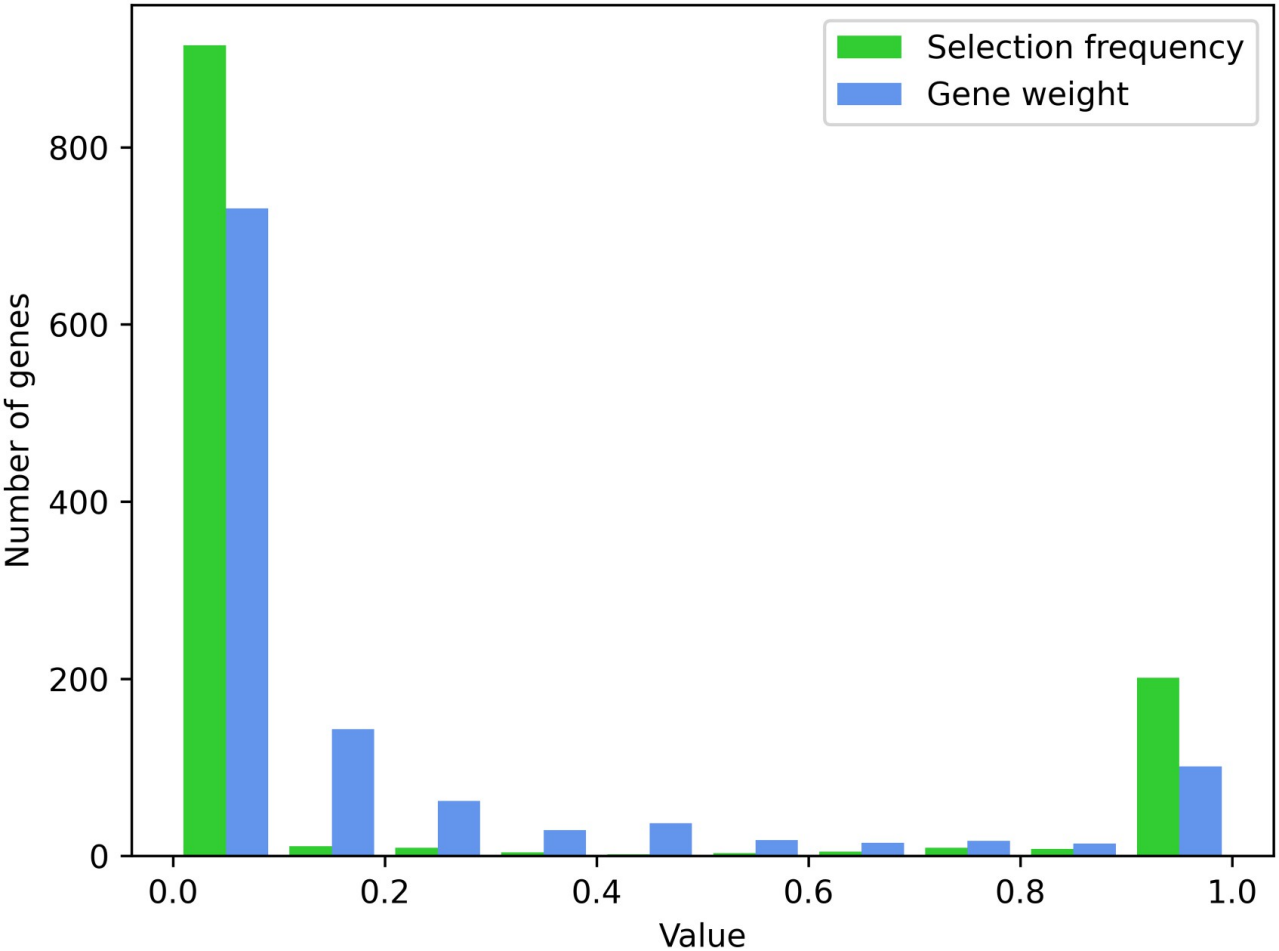
Selection frequency: Fraction of SigNet runs where a gene was selected as the active gene within its locus, averaged over 100 runs. Gene weight: Bayesian scores expressed as gene weights, as defined by Eq (41), averaged over final values from the same 100 runs.

Final active networks were obtained for 100 independent runs starting from a ‘best guess’ initialization favoring genes with stronger functional evidence and defaulting to the minimum distance gene in loci without functional evidence. The runs required a median of 6 passes to converge, and the final active genes were identical across all 100 runs for 210 of the 245 loci. Frequencies of selected genes are therefore strongly peaked at 0 and 1 (Fig 2). Only 51 of the 1167 genes in GWAS loci had selection frequencies between 0.1 and 0.9. The gene weights defined by Eq (41) are similarly peaked at 0 and 1. Selecting the most likely gene at each locus causes the selection frequencies to be peaked more strongly than the gene weight; a Gibbs sample would give a selection frequency more similar to the gene weight. Results from these runs are available as S1 Table with table columns defined in S2 Table.

Since a major purpose of a Gibbs sampler is to explore more regions configuration space, we assessed the importance of the initial configuration by performing 100 runs with the initial active genes selected uniformly at random within each locus, rather than the best guess initialization. Compared with the genes that were selected for the 100 runs with the best guess initialization, the same gene was selected at 232 of the 245 loci. For the remaining 13 loci, the random initialized networks select the same gene as the candidate gene of the best guess initialization but for less than half of the runs. We used best guess initialization thereafter because it gave faster convergence.

Final parameter values had little dispersion across the 100 independent runs (Table 5). The distance parameter increased from 148.0 kb from the ‘best guess’ initialization, which selected the closest gene at information-poor loci, to a final value of 161.3 ± 0.9 kb. The Mendelian and Exome scores show no dispersion because the numbers of Mendelian and Exome genes selected were identical at the end of each run. The exponentials of the scores for special features can be interpreted as odds for selecting a gene with the feature, other evidence being equal: 15× for Mendelian evidence, 55× for Exome evidence, and 11× for Colocalization evidence. While the lower odds for Mendelian evidence may be surprising, the explanation is simple: two loci had two Mendelian genes each, preventing two Mendelian genes from being selected and lowering the Mendelian score (see below).

**Table 5:** Parameter values.

Parameter	Initial value, full data	Final value, full data	Final value, 80% subsets
*γ*	148.0 kb	161.3 ± 0.9 kb	158.8 ± 10 kb
*S*_Mendelian_	2.7	2.7 ± 0.0	2.6 ± 0.3
*S*_Exome_	4.0	4.0 ± 0.0	3.7 ± 0.2
*S*_Coloc_	2.7	2.4 ± 0.1	2.4 ± 0.2

Initial values are from ‘best guess’ selection of the active gene at each locus. Final values for the full data provide the standard deviation for 100 independent runs with random restarts. Final values for 80% subsets provide the standard deviation for performing one run on each of 100 subsets of 80% of the loci selected uniformly at random.

We examined the robustness of these estimated parameters by running SigNet once on each of 100 subsets of 80% of the loci, selected uniformly at random, and comparing the final parameters with the values obtained for the 100 random restarts using the full data (Table 5). The distance parameters agree within the sampling standard deviation, as do the scores for Mendelian genes and co-localized genes. The score for genes with exome evidence are smaller for the 80% subsets, 3.7±0.2 versus 4.0±0.0 for the full data. This difference is due to our use of pseudocounts, which bias the scores towards 0 for smaller data sets. In the full data of 226 GWAS loci, 1167 total genes, and 12 genes with exome evidence, each exome gene was always selected (see GWAS loci with exome-chip or colocalization evidence). The exome score for the full data was therefore ln[(12 + 1)/(0 + 1)] − ln[(214 + 1)/(941 + 1)] = 4.04. If all gene counts are reduced proportionally in the 80% subsets, we expect an exome score of approximately ln[(9.6 + 1)/(0 + 1)]−ln[(171.2 + 1)/(752.8 + 1)] = 3.84. Thus, reducing from the full data to 80% of the data accounts for much of the difference between the full data and the smaller subsets. We separately examined the ability to recover genes whose functional information was hidden; see Importance of functional information.

We next examined the selection of genes based on functional evidence, analyzed in terms of loci (Table 6) and in terms of genes (Table 7). If a locus has a gene with functional evidence, that gene is usually selected. Of the 56 genes with any functional evidence (highest evidence 10 Mendelian, 8 exome, 28 colocalized), 44 were the selected gene. Of the 12 genes with functional evidence that were not selected, 10 were in loci where the selected gene did have functional evidence. There were only two cases in which a gene with functional evidence (in both cases colocalization) was passed over in favor of a gene without functional evidence. At a locus where *VPS29* was colocalized, *ATP2A2* (the minimum distance gene) was selected, and at a locus where *DDX17* was colocalized and *SUN2* was the minimum distance gene, *JOSD1* was selected.

**Table 6:** Selection of genes by level of information at the GWAS locus.

Highest level of information in locus	Number of loci	Number of genes selected				
		Mendelian	Exome	Coloc	Mindist	None
Mendelian	10	10	0	0	0	0
Exome	8	-	8	0	0	0
Coloc	28	-	-	26	1	1
None	180	-	-	-	124	56
**Total**	226	10	8	26	125	57

Number of genes selected: the selected gene is counted once in the category corresponding to the highest level evidence in the order Mendelian, Exome, Colocalization, MinDist, and None. Thus a gene that is Mendelian and colocalized is counted in the Mendelian column.

**Table 7:** Selection of genes by level of information for the gene.

Highest level of information for gene	Number of genes	This gene selected	Other gene selected				
			Mendelian	Exome	Coloc	Mindist	None
Mendelian	12	10	2	0	0	0	0
Exome	8	8	0	0	0	0	0
Coloc	36	26	1	1	6	1	1
Mindist	200	125	3	2	13	0	57
None	911	57	73	58	174	206	343

Other gene selected: the selected gene is counted once in the category corresponding to the highest level evidence in the order Mendelian, Exome, Colocalization, MinDist, and None. Thus a gene that is Mendelian and colocalized is counted in the Mendelian column.

**Fig 3:**
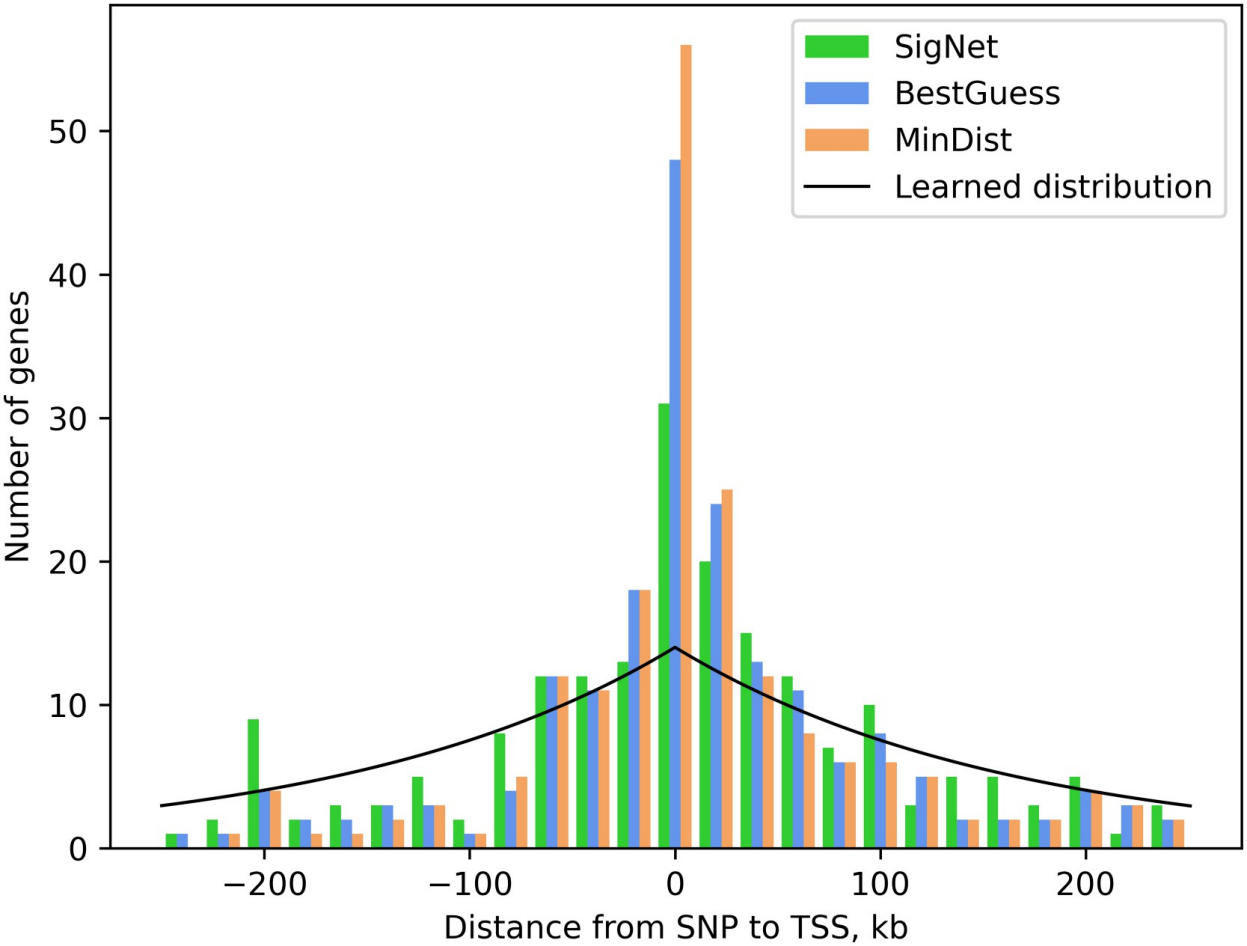
Distribution of the signed distance from a GWAS SNP to the transcription start site of the active gene selected at each locus. Distributions are shown for genes selected by a minimum distance criterion, by best guess initialization, and by SigNet. Learned distribution: exponential distribution with converged distance parameter 161.3 kb used by SigNet.

Finally, we examined selection of genes as a function of distance from the closest GWAS SNP (Fig 3), comparing genes selected by minimum distance to the SNP, by best guess initialization, and by SigNet. Signed distances were calculated relative to the transcription start site, using the maximal gene boundary for genes with multiple reported transcription starts. Negative distances correspond to SNPs that are located 5′ relative to the start site on the sense strand. Genes with exome evidence usually have closest SNPs within the gene body, corresponding to positive distances. In general, all three distributions are peaked at distance 0 and then decrease. Best guess and SigNet have distributions that are shifted somewhat more outward, with functional evidence favoring more distant genes over the minimum distance gene. The learned distribution decays less rapidly because of GWAS loci in gene deserts, where the minimum distance gene may be quite far from the SNP.

To avoid losing genes with strong functional evidence because of other strong nearby candidates, we augmented the single gene selected by SigNet at each locus with any additional genes with functional evidence that were not selected. We term this method SigNet+. The SigNet and SigNet+ results are identical for the 37 loci with a single gene with functional evidence (which is also the selected gene for 35 of these loci) and for the 179 information-poor loci with no genes with functional evidence. The results differ, however, at the 14 loci with multiple genes with functional evidence, with SigNet selecting one of these genes at each of the loci. Methods that permit multiple genes to be selected at a locus are possible (see Discussion), but often involve optimization of hyper-parameters.

### Importance of functional information

To test the importance of functional information from Mendelian studies, exome variation, and colocalization, we performed tests in which these annotations were hidden (Table 8). We considered the 37 loci where only a single gene had Mendelian, exome, or colocalization data. We then performed a series of 37 tests in which the functional information for one of these genes was hidden, 100 runs were performed using the best-guess initialization, and the gene selected most often at the locus in question was determined. For all 7 genes with Mendelian information, the Mendelian gene was recovered even with its information hidden. For genes with exome variation, 3 of 7 were recovered, and for genes with colocalization information, 12 of 23 were recovered. Two of the colocalized genes not recovered, *DDX17* and *VPS29*, were also not recovered when colocalization information was provided (see below, GWAS loci where multiple genes may be causal).

**Table 8:** Number recovered, using information: Number of genes in the specified category selected by SigNet most often over 100 runs. Number recovered, hiding information: Number of genes in the specified category selected by SigNet, hiding the functional information and performing 100 runs for each of the 37 genes in turn.

Highest level of information for gene	Number of genes	Number recovered	
		Using information	Hiding information
Mendelian	7	7	7
Exome	7	7	3
Coloc	23	21	12

Of the 7 genes with exome information that were tested, the 3 recovered in the runs with information hidden were *NRAP*, *RNF207*, and *TTN*. The genes not recovered were *NACA* (nonsynonymous variant, *GLS2* selected instead), *PM20D1* (nonsynonymous variant, *SLC41A1* selected instead and also an eQTL target of the variant), *SENP2* (nonsynonymous exome variant, *LIPH* selected instead), and SLC12A7 (synonymous splicing variant, *NKD2* selected instead and also an eQTL target of the variant).

This analysis indicates the importance of integrating multiple types of information. Recovery of Mendelian genes may be better than other categories because these genes may be better studied, with more protein interaction data available. Also, while Mendelian genes are trustworthy as a gold standard for causality, genes with exome or colocalization data are less certain to be the true causal gene at a locus.

### SigNet genes improve pathway enrichment over closest genes and shuffled networks

A default approach to select the most likely causal gene at a GWAS locus is to select the gene whose transcription start site is closest to a GWAS SNP, here termed the minimum-distance (MinDist) method. Of the 226 loci, SigNet and MinDist agree at 149 loci, or 66%. Of the remaining 77 loci where the MinDist gene is not selected, 20 had within-locus information contributing to the selection of the causal gene: 1 had only Mendelian evidence, 3 had only exome evidence, 13 had only colocalization evidence, 2 had both Mendelian and exome evidence, and 1 had Mendelian, exome, and colocalization evidence. The candidate causal gene at the remaining 57 loci, or 25% of the total loci, were selected based primarily on network connectivity with genes selected by SigNet at other loci.

Of the 226 loci, 46 have functional evidence. Of the loci with functional evidence, SigNet and MinDist agree at 25, and SigNet selects a more distant gene that has functional evidence at 21 loci. The number of information-poor loci, lacking strong functional evidence, is 180, or 80% of the total. Among the information-poor loci, SigNet and MinDist agreed at 124 loci and SigNet selected a more distant gene at 56 loci.

Pathway enrichment provides an assessment of the relative performance of gene selection by SigNet, SigNet+, and MinDist. The Enrichr method [43–45] was used to calculate p-values for pathways from KEGG [46]. The SigNet method performs better than selecting the minimum distance gene for significant cardiovascular-related pathways (Tables 9 and 10). Including multiple genes with functional evidence with SigNet+ improves the number of pathway genes for the ‘Adrenergic signaling in cardiomyocytes’, ‘Circadian entrainment’, and ‘Oxytocin signaling’ pathways.

**Table 9:** Cardiovascular pathway enrichment, all 245 loci.

Pathway	Number of genes							
	Pathway	SigNet+	SigNet	BestGuess	MinDist	Shuffled	AFib	Cardio
Adrenergic signaling in cardiomyocytes	150	19	18	15	14	15.0 ± 1.1	16	18
Arrhythmogenic rt ventricular cardiomyopathy	77	10	10	9	9	8.7 ± 0.7	10	11
Cardiac muscle contraction	87	11	11	7	7	7.6 ± 0.7	9	9
Cholinergic synapse	113	11	11	9	6	8.7 ± 0.9	5	7
Circadian entrainment	97	13	12	12	10	11.0 ± 0.9	8	10
Dilated cardiomyopathy	96	9	9	6	6	6.4 ± 0.6	10	9
GnRH signaling pathway	93	7	7	6	5	6.2 ± 0.4	7	8
Hypertrophic cardiomyopathy	90	11	11	8	8	8.1 ± 0.8	11	10
Oxytocin signaling pathway	154	14	14	12	11	12.7 ± 0.8	13	15

Overlap of genes selected by different methods with genes in cardiovascular pathways. SigNet+ and SigNet: genes selected most often at each locus over 100 independent runs. BestGuess: genes selected by best guess initialization based on functional information and distance from GWAS SNP. MinDist: genes selected by minimum distance to GWAS SNP. Shuffled: genes selected using shuffled networks using degree-preserving randomization [47, 48], with standard deviation over 100 independently shuffled networks. AFib and Cardio: genes selected by maximum polygenic priority score (PoPS) at each locus for atrial fibrillation (AFib) and cardiovascular disease (Cardio) phenotypes [23].

**Table 10:** Cardiovascular pathway enrichment, all 245 loci.

Pathway	p-value						
	SigNet+	SigNet	BestGuess	MinDist	Shuffled	AFib	Cardio
Adrenergic signaling in cardiomyocytes	7.5 × 10^−14^	3.9 × 10^−13^	5.1 × 10^−10^	4.8 × 10^−9^	5.0 × 10^−11^	5.0 × 10^−11^	3.9 × 10^−13^
Arrhythmogenic rt ventricular cardiomyopathy	5.3 × 10^−8^	3.4 × 10^−8^	4.2 × 10^−7^	4.2 × 10^−7^	4.2 × 10^−7^	3.4 × 10^−8^	2.4 × 10^−9^
Cardiac muscle contraction	1.5 × 10^−8^	9.2 × 10^−9^	9.7 × 10^−5^	9.7 × 10^−5^	1.2 × 10^−5^	1.2 × 10^−6^	1.2 × 10^−6^
Cholinergic synapse	2.3 × 10^−7^	1.4 × 10^−7^	1.1 × 10^−5^	2.6 × 10^−3^	1.3 × 10^−6^	1.2 × 10^−2^	4.9 × 10^−4^
Circadian entrainment	3.4 × 10^−10^	2.5 × 10^−9^	2.5 × 10^−9^	3.1 × 10^−7^	2.9 × 10^−8^	2.6 × 10^−5^	3.1 × 10^−7^
Dilated cardiomyopathy	4.1 × 10^−6^	2.8 × 10^−6^	1.2 × 10^−3^	1.2 × 10^−3^	1.2 × 10^−3^	2.8 × 10^−7^	2.8 × 10^−6^
GnRH signaling pathway	2.0 × 10^−4^	1.5 × 10^−4^	9.9 × 10^−4^	5.7 × 10^−3^	9.9 × 10^−4^	1.5 × 10^−4^	1.9 × 10^−5^
Hypertrophic cardiomyopathy	2.2 × 10^−8^	1.3 × 10^−8^	1.5 × 10^−5^	1.5 × 10^−5^	1.2 × 10^−4^	1.3 × 10^−8^	1.5 × 10^−7^
Oxytocin signaling pathway	1.3 × 10^−8^	6.8 × 10^−9^	4.5 × 10^−7^	3.2 × 10^−6^	5.8 × 10^−8^	5.8 × 10^−8^	7.4 × 10^−10^

Statistical significance of overlap of genes selected by different methods with genes in cardiovascular pathways. SigNet+ and SigNet: genes selected most often at each locus over 100 independent runs. BestGuess: genes selected by best guess initialization based on functional information and distance from GWAS SNP. MinDist: genes selected by minimum distance to GWAS SNP. Shuffled: genes selected using shuffled networks using degree-preserving randomization [47, 48], with geometric mean of p-values over 100 independently shuffled networks. AFib and Cardio: genes selected by maximum polygenic priority score (PoPS) at each locus for atrial fibrillation (AFib) and cardiovascular disease (Cardio) [23] phenotypes.

To assess the improvements that were due to the network data, we also performed tests excluding the network data and using shuffled versions of the network data. We ran SigNet on 100 shuffled versions of the protein-protein and genome-regulatory interactions networks. Each shuffled network was generated to maintain the vertex degree of each gene in the actual network [47], using the implementations configuration_model for protein-protein interactions and directed_configuration_model for gene-regulatory interactions from NetworkX [48]. We again used pathway enrichment to assess performance. The results using the true network interactions were better than the results using shuffled networks (Tables 9 and 10). We also ran SigNet without using any network data. Gene selection without network data was identical to the best guess initialization, which performs similarly to gene selection with randomized network data. Equivalent performance without network data and with randomized network data is ideal performance for a Bayesian method and suggests that SigNet is not overfitting the network data.

### SigNet compares favorably with polygenic priority scores

We also compared our gene selection method with polygenic priority scores from the PoPS method [23], which provides a compendium of pre-calculated scores for phenotypes with ample GWAS data. Rather than using GWAS results directly, this method builds a regression model for GWAS data from extensive genomic and proteomic data, then reports the model output as the score. We used scores from the PoPS_FullResults.txt file for the two most relevant phenotypes, atrial fibrillation (AFib) and cardiovascular disease (Cardio). Some differences may arise because SigNet used data from the GWAS Catalog [17], whereas PoPS used data from UK BioBank. The cohorts are both European ancestry, however, and the cohort size used by PoPS was larger, with 349,512 individuals for AFib and 408,963 individuals for Cardio, whereas our GWAS results are from cohorts of 60,255 to 134,251 individuals (Table 1). Therefore, differences in cohorts are likely to favor PoPS.

Of the 1195 genes within our loci, PoPS scores were reported for 1069. The difference in count of 126 genes arises from updates to gene names and genome annotations between the GRCh38 version we used and the earlier version used by PoPS. These genes were dropped from comparisons. Additionally, some genes in the GRCh38 version we used map to multiple genes in the version used by PoPS, with distinct scores for each gene. A summary table joins SigNet results with PoPS results (S3 Table), with empty cells for the missing genes and multiple rows for the duplicated genes. To make score more comparable, we calculated a locus-specific baseline for each method as the maximum score for a gene in that locus. We then subtracted this baseline from all genes within a locus, giving the best gene for each method a baseline-subtracted score of 0 and other genes increasingly negative scores. For a robust comparison, we also converted scores to integer ranks in descending order of scores within each locus.

**Fig 4:**
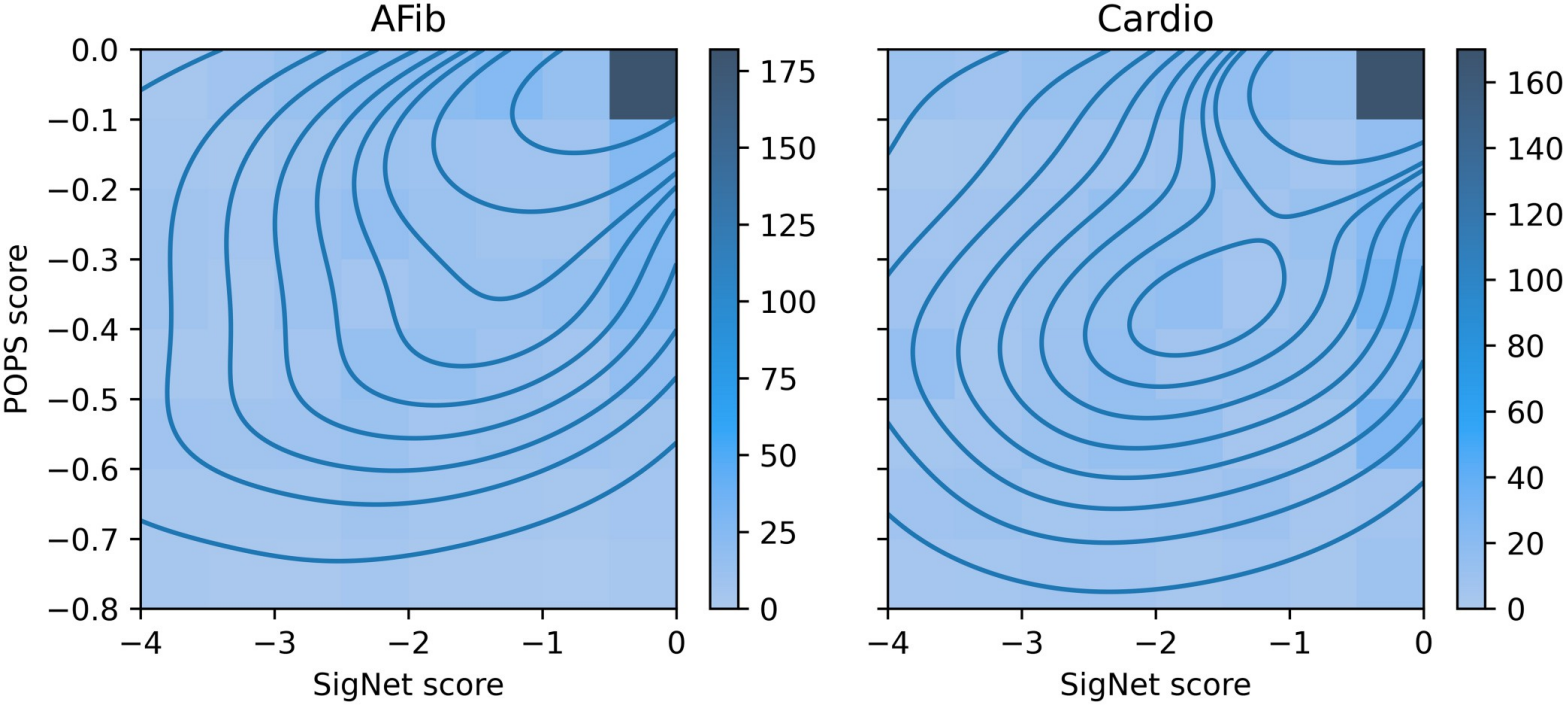
Density plot of gene scores from SigNet compared with PoPS for the PoPS phenotypes AFib (left) and Cardio (right). More saturated colors indicate higher density, with contour lines from kernel density estimation.

The SigNet and PoPS baseline-subtracted scores and ranks are highly correlated, with density plots of scores showing maximum density when both methods score the same gene at or near the top (Fig 4). For the AFib phenotype, the Pearson correlation of scores is 0.46 (p-value 2.5 × 10^−56^) and the Spearman correlation of ranks is 0.53 (p-value 6.9 × 10^−101^). For the Cardio phenotype, the score correlation is smaller but still significant, 0.15 (p-value 4.2 × 10^−7^), and the rank correlation is 0.47 (p-value 1.2 × 10^−89^). The AFib and Cardio results from PoPS are themselves significantly correlated, a score correlation of 0.43 (p-value 1.4 × 10^−51^) and a rank correlation of 0.72 (p-value 3.3 × 10^−179^).

We then examined concordance of genes ranked first or second within a locus (Table 11). The top-ranked gene from SigNet agrees with top-ranked PoPS gene at 138 loci for the AFib phenotype and 140 loci for the Cardio phenotype. The overlap increases to 211 genes (86% of the 245 loci) for genes ranked first by one method and first or second by the other method for AFib, and to 202 genes (82% of loci) for Cardio.

**Table 11:** Comparison of SigNet and PoPS gene rankings.

SigNet Rank	PoPS AFib Rank			PoPS Cardio Rank		
	1	2	≥ 3	1	2	≥ 3
1	138	36	60	140	34	60
2	37	34	69	28	34	78
≥ 3	68	77	588	75	79	579

Because ground truth is not yet available for GWAS data, we used pathway enrichment as a proxy for comparing the number of pathway genes recovered by each method (Table 9) and the corresponding p-values (Table 10). Comparing SigNet with PoPS for the AFib phenotype, SigNet finds more genes for 5 of the pathways and fewer genes in 1 pathway of the 9 cardiovascular pathways (Table 9). Comparing with PoPS for the Cardio phenotype, SigNet find more genes for 4 pathways and fewer genes for 3 pathways. SigNet performs better in each case, nearly reaching statistical significance for AFib (paired two-sided t-test for number of genes recovered, p-value = 0.071 for AFib and p-value = 0.28 for Cardio).

We investigated the differences in genes recovered where functional evidence favors clear candidate genes within a locus (Table 12). Of the 18 GWAS loci with Mendelian or exome evidence for at least one gene (excluding the 19 Mendelian loci without GWAS evidence), SigNet recovers a Mendelian or exome gene in each (see below, Sec. GWAS loci with Mendelian evidence and GWAS loci with exome-chip or colocalization evidence). Within these 18 loci with strong evidence, the PoPS results for AFib and Cardio both select a gene not included in our Mendelian or exome evidence at 7 loci.

**Table 12:** Genes with strong functional evidence found by SigNet but not by PoPS.

SigNet		PoPS	
Gene	Evidence	AFib	Cardio
*RNF207*	Exome	*ACOT7*	*PLEKHG5*
*PM20D1*	Exome, MinDist	*NUCKS1*	*NUCKS1*
*SLC4A3*	Medelian, Exome	*DES*	*DES*
*CASR*	Exome	*FAM162A*	*KPNA1*
*KCNQ1*	Mendelian, Exome	*INS*	*INS*
*KCNJ5*	Mendelian, Colocalized, MinDist	*FLI1*	*FLI1*
*KCNJ2*	Mendelian, MinDist	*KCNJ16*	*KCNJ16*

In one of these loci where SigNet and PoPS differ, both selected genes may be causal: at the locus where SigNet selects *SLC4A3*, responsible for a Mendelian form of short QT syndrome, PoPS selects *DES* (desmin), responsible for a Mendelian form of cardiomyopathy [3]. Other genes selected by PoPS include *FLI1*, a transcriptional regulator of blood and endothelial development [49], in a locus where SigNet selected *KCNJ5*, responsible for a Mendelian form of long QT syndrome [3], and *KCNJ16*, a potassium channel responsible for deafness [3], where SigNet selected *KCNJ2*, a different potassium channel responsible for Mendelian forms of atrial fibrillation and short QT syndrome [3]. These results suggest that SigNet performs better than PoPS at loci with strong functional evidence.

### GWAS loci with Mendelian evidence

At many loci, functional evidence points to a gene other than the MinDist gene as the causal gene. The SigNet method is effective in using this information to select the appropriate gene. Mendelian evidence is particularly strong. Of the 12 Mendelian genes in the GWAS loci, 5 were not selected by MinDist. Of these, the genes *KCNE2* and *SCN10A* occur in loci with two Mendelian genes, which were selected instead. Of the remaining three genes, *CACNA1C*, *KCNQ1*, and *SLC4A3*, all were selected by SigNet but not MinDist. The genes are each considered in turn.

**Fig 5:**
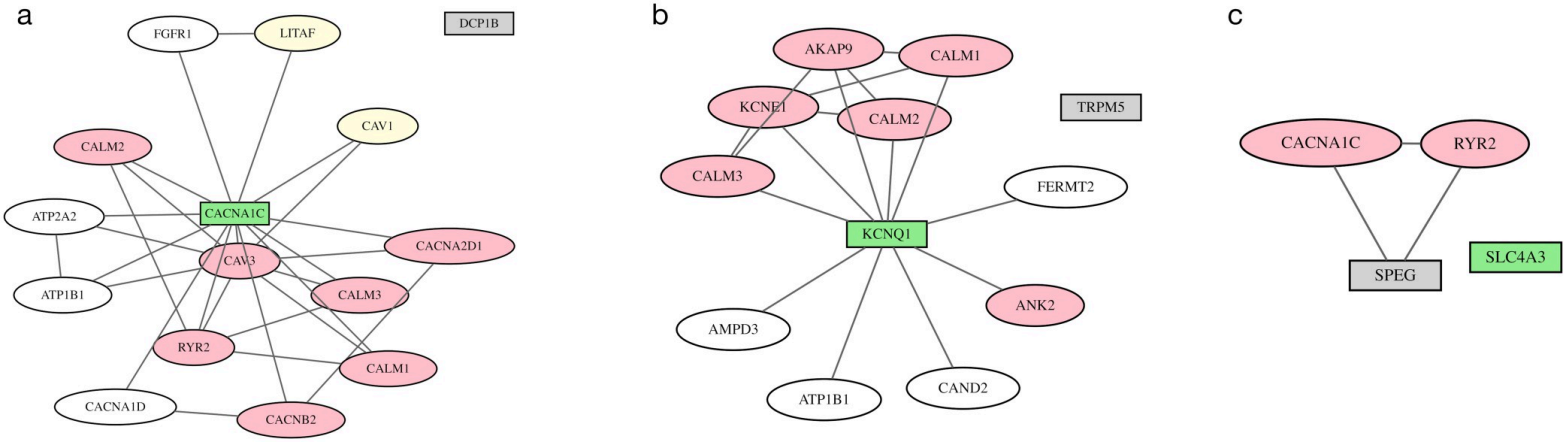
GWAS loci with Mendelian evidence. SigNet selects the gene with Mendelian evidence (green rectangle) over the closest gene in the locus to a GWAS SNP (gray rectangle). Pink ovals represent genes with Mendelian evidence; yellow ovals represent colocalized genes; white ovals represent information-poor genes; and gray lines represent protein-protein interactions. Networks are shown for three individual loci, highlighting the gene selected by SigNet: (a) *CACNA1C*, (b) *KCNQ1*, (c) *SLC4A3*.

The Mendelian gene *CACNA1C* is 104,528 bp from a locus on chromosome 12 defined by SNP rs2283274 at position 2075300, whose 250 kb flanks include one other protein-coding genes: *DCP1B* at 70,765 bp distance. The MinDist gene, *DCP1B*, encodes mRNA-decapping enzyme 1B, which has no literature reports suggesting involvement with cardiovascular phenotypes. The Mendelian gene selected by SigNet, CACNA1C, encodes a calcium voltage-gated channel that is the target of calcium channel blockers (Fig 5).

The Mendelian gene *KCNQ1* is at a locus on chromosome 11 defined by rs2074238, rs2301696, and rs7122937, all from the QT GWAS (Fig 5). The gene selected by MinDist is *TRPM5*, at 1,239 bp from rs2301696. The *KCNQ1* gene is the closest to rs2074238 at 18,889 bp; its gene product is a potassium voltage gated channel with alleles responsible for hereditary forms of long QT syndrome. The gene *TRPM5* is not directly related to cardiovascular function. Instead, it is implicated in taste transduction. The activation/impairment of *TRPM5* has been shown to reduce/increase salt-induced cardiovascular function [50, 51]. *TRPM5* has no interaction partners in the network selected by SigNet. These results suggest that there is a single causal gene at this locus, *KCNQ1*.

The Mendelian *SLC4A3* gene, which also has exome evidence, is at a locus on chromosome 2 defined by rs55910611 and rs907683 associated with heart rate, JT, and QT phenotypes (Fig 5). This locus contains 23 genes, all of which are protein-coding. While *SLC4A3* is the closest gene to rs55910611 at 8,296 bp distance, the MinDist gene is *SPEG*, 24 bp from rs907683, and also colocalizing with this SNP. The SLC4A3 protein is a plasma membrane anion exchange protein with mutations responsible for short QT syndrome and elevated risk of ventricular fibrillation and sudden cardiac death [52]. The *SPEG* gene encodes a myosin light chain kinase and regulator of cardiac calcium homeostasis with mutations causing dilated cardiomyopathy, atrial fibrillation, and heart failure [53]. Furthermore, SPEG interacts with CACNA1C and RYR2, both selected by SigNet. Strong evidence for both *SLC4A3* and *SPEG* suggests that this locus contains multiple causal genes.

### GWAS loci with exome-chip or colocalization evidence

All of the 12 genes that were implicated in a recent exome-chip study of individuals with ventricular repolarization [35] were selected by SigNet. Of these twelve genes, four genes (*KCNH2*, *KCNQ1*, *SCN10A*, *SLC4A3*) also had Mendelian evidence, and are thus accounted for as Mendelian genes. The remaining eight genes that had exome-chip evidence, were also selected by SigNet (Table 7). Of these eight genes, three were not the minimum distance gene of loci.

**Fig 6:**
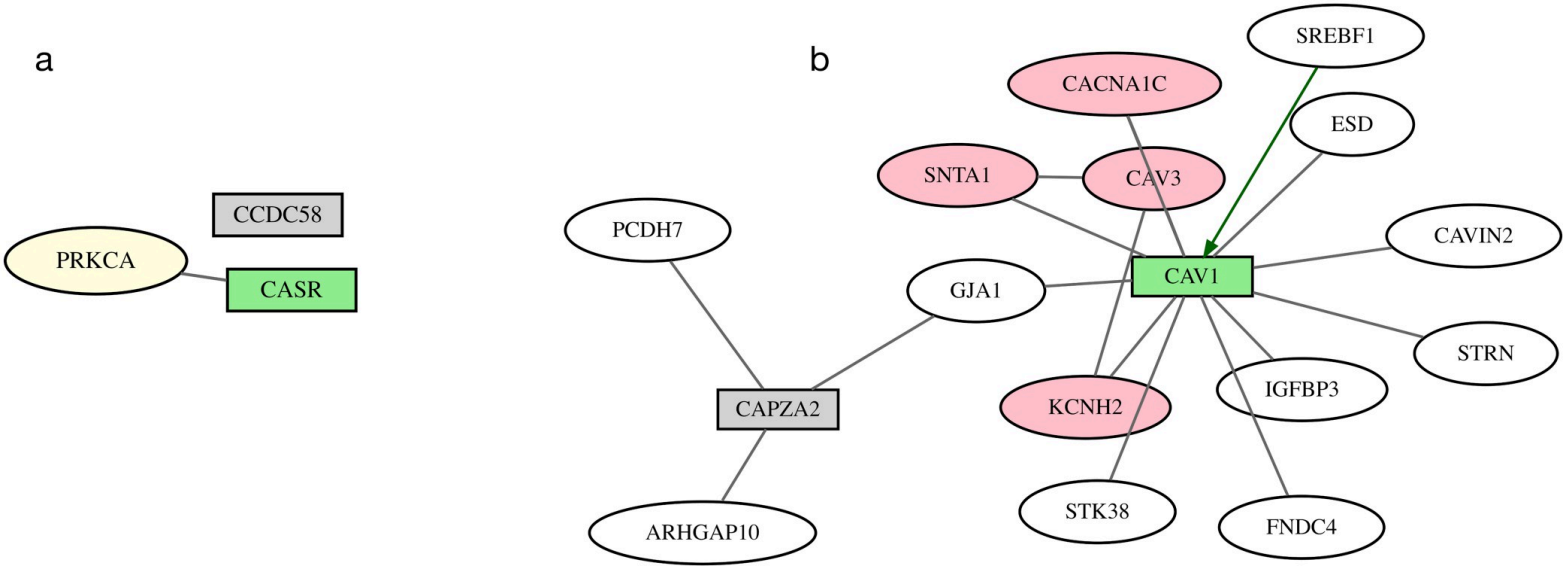
GWAS loci with exome-chip or colocalization evidence. SigNet selects the gene with exome-chip or colocalization evidence (green rectangle) over the closest gene in the locus to a GWAS SNP (gray rectangle). Pink ovals represent genes with Mendelian evidence; white ovals represent information-poor genes; gray lines represent protein-protein interactions; and green arrow represents gene-regulatory interaction. Networks are shown for two individual loci, highlighting the gene selected by SigNet: (a) *CASR*, (b) *CAV1*.

For example, in the locus defined by rs11920570 and rs1801725 associated with HR, JT, and PR phenotypes, SigNet selected the *CASR* gene, which has exome evidence, rather than the minimum distance gene, *CCDC58* (Fig 6). The SNP rs1801725 is in the terminal exon of *CASR*, located 101 kb downstream from the transcriptional start site. *CASR* is expressed in various cardiovascular cell types and has a crucial role in cardiovascular diseases [54]. The distance from *CCDC58* to rs11920570 is much smaller, only 12 kb. While it is colocalized with HR, there are no studies connecting this gene to cardiovascular disease.

For the 28 loci that have a colocalized gene as their highest level of information, 26 selected genes were the genes that had colocalization evidence. Of these, 14 were not the minimum distance gene of the locus. An example is the *CAV1* gene, which is colocalized with PR is at a locus on chromosome 4 defined by rs3807989, rs41748, and rs9920, identified in GWAS studies for the HR, PR, and QT intervals. The minimum distance gene of this locus *CAPZA2*, which is located 4,551 bp from the locus was not selected by SigNet. Instead, *CAV1* which is located 21,193 bp from the locus was selected. The CAV1 protein forms interactions with the protein product of eleven other selected genes in the network and a gene-regulatory interaction (Fig 6). The deletion of *CAV1* in mice diminishes caveolae formation, resulting in cardiac defects [55–57]. Similarly, Zebrafish lacking *CAV1* showed impaired cardiac function [58]. *CAPZA2* caps the barbed ends of actin filaments. While it is expressed in many tissues including the heart, there is less evidence linking this gene to cardiovascular disease.

### GWAS loci with no functional evidence

The SigNet method was designed to use cross-locus information to improve the selection of causal genes at loci lacking within-locus functional evidence. We present several loci where the genes selected by SigNet and MinDist are different, and where network connectivity with genes selected at other loci strongly suggests that the SigNet prediction of the causal gene is correct.

**Fig 7:**
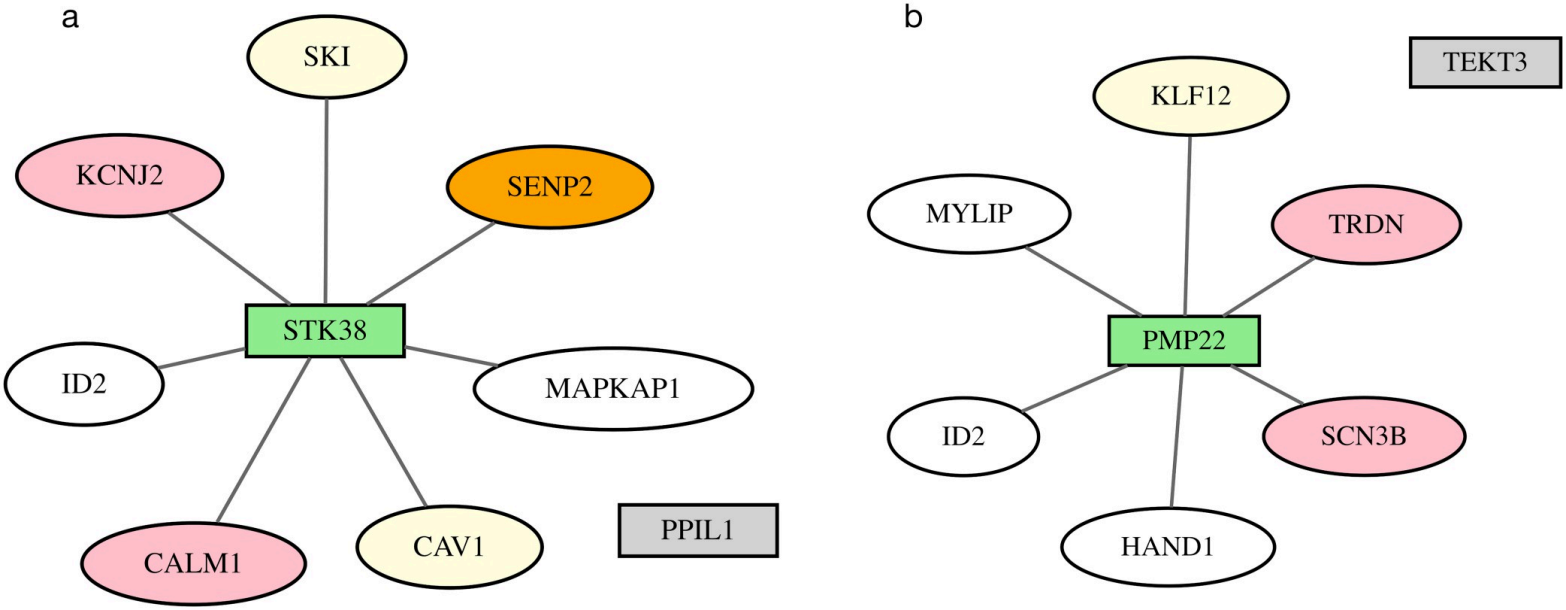
GWAS loci with no functional evidence. SigNet selects the gene (green rectangle) based on network connectivity with genes selected at other loci, over the closest gene in the locus to a GWAS SNP (gray rectangle). Pink ovals represent genes with Mendelian evidence; orange ovals represent exome-chip evidence; yellow ovals represent colocalized genes; white ovals represent information-poor genes; and gray lines represent protein-protein interactions. Networks are shown for loci containing (a) *STK38*, (b) *PMP22*.

The *STK38* gene is selected by SigNet at a locus on chromosome 6 defined by SNPs rs1321311, rs236349, and rs9470361 (Fig 7). The *STK38* gene is 107,644 bp from rs1321311. The MinDist gene is *PPIL1*, located 2,038 bp from rs236349. STK38 modulates the stability of Rbm24 protein [59], which is a key regulator in cardiogenesis [60]. STK38 forms protein-protein interactions with CALM1, CAV1, ID2, KCNJ2, MAPKAP1, SENP2, and SKI in the network. The MinDist gene, *PPIL1*, encodes a peptidylprolyl isomerase that may function in spliceosome activity and protein folding. It has no interaction partners in the selected network and no substantial literature reports suggesting relevance to cardiac electrophysiology.

The *PMP22* gene is selected by SigNet at a locus on chromosome 17 defined by the SNP rs79121763, identified in the heart rate GWAS, and 19,670 bp from the GWAS SNP (Fig 7). The minimum distance gene is *TEKT3*, 11,84 bp away from the SNP. The PMP22 protein has physical interactions with proteins encoded by genes selected at 7 other loci, whereas TEKT3 has no interactions with selected genes. The *PMP22* gene encodes peripheral myelin protein-22. This may be a novel candidate gene at the locus.

### GWAS loci where multiple genes may be causal

As discussed earlier, there were a total of 12 genes with function evidence that were not selected by SigNet. Of these 12, 10 were in loci where the selected gene also had functional evidence. Examination suggests that these loci contain multiple causal genes. To prevent the exclusion of genes with strong functional evidence due to other strong nearby candidates, we augmented the selection of a single gene made by SigNet at each locus to include any additional genes supported by functional evidence that were not initially chosen (SigNet+).

**Fig 8:**
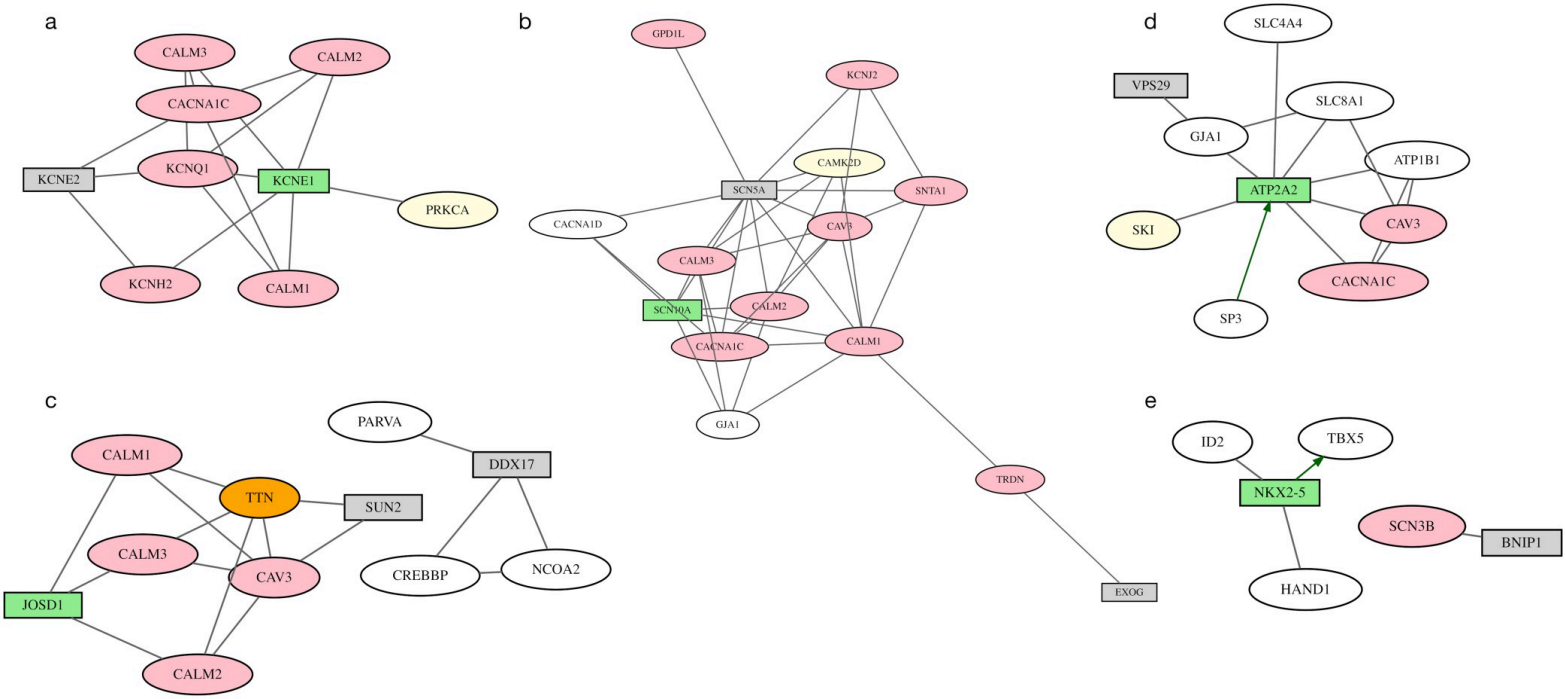
GWAS loci where multiple genes may be causal. SigNet selects the gene (green rectangle) based on within locus and across loci evidence. SigNet+ augments the selection with other genes in the locus that have functional evidence (gray rectangle). Pink ovals represent genes with Mendelian evidence; orange ovals represent exome-chip evidence; yellow ovals represent colocalized genes; white ovals represent information-poor genes; gray lines represent protein-protein interactions; and green arrows represent gene-regulatory interactions. Networks are shown for loci containing (a) *KCNE1*, (b) *SCN10A*, (c) *JOSD1*, (d) *ATP2A2*, (e) *NKX2–5*.

Of the 12 genes with Mendelian evidence, presumably the strongest level of evidence, 2 were not selected: *KCNE2* and *SCN5A*. Each is in a GWAS loci that contains an additional Mendelian gene that was selected instead. One locus contains Mendelian genes *KCNE1* and *KCNE2*, and a second locus contains Mendelian genes *SCN5A* and *SCN10A*. Local networks show dense interactions between the Mendelian genes at the *KCNE1*-*KCNE2* locus (Fig 8) and the *SCN5A*-*SCN10A* locus (Fig 8) and the genes selected at other loci.

Similarly, at two loci with colocalization evidence, SigNet selects a non-colocalized gene (Table 6), and evidence suggests that both the colocalized gene and the selected gene may be causal. Colocalized gene *DDX17* is 246,785 bp from rs2076028, identified in the GWAS studies for HR [34] (Fig 8). Instead of selecting this gene, however, SigNet selected *JOSD1*, which is 52,889 bp away from the SNP. The JOSD1 protein interacts with genes selected at other loci, which are themselves highly connected to other genes. These interactions cause *JOSD1* to be selected, even though *DDX17* has colocalization and interaction evidence. *DDX17* has been identified as a binding partner of CPhar, which regulates the expression of proliferation markers, in cultured neonatal mouse cardiomyocytes [61] The gene closest to the SNP is *SUN2*, 21,155 bp away. Loss of this gene causes cardiac hypertrophy in mice [62]. Thus, this locus may contain multiple causal genes, all of which are listed in SigNet+ and the selected gene, *JOSD1* may be a novel candidate gene at the locus.

The *VPS29* gene is at a locus on chromosome 12 defined by rs11068997, rs3026445, and rs75714509 identified in GWAS studies for the QT phenotype and is colocalized with QT (Fig 8). *VPS29* is located 140,181 bp from the locus and was not selected. Instead SigNet selects the gene *ATP2A2*, which is the minimum distance gene located 4,642 bp from the locus. *ATP2A2* forms a gene-regulatory interaction (green arrow) with a selected gene of another locus, as well as seven protein-protein interactions with other selected genes, two of which have Mendelian evidence. The *ATP2A2* gene encodes SERCA2, which controls the cardiac contraction-relaxation cycle by regulating Ca2+ uptake levels [63, 64].

Loci without functional evidence may also contain multiple causal genes. An example is the locus containing *NKX2–5* and *BNIP1* (Fig 8). The *NKX2–5* gene occurs at a locus defined by rs4868243 from a heart rate GWAS [34] and rs255292 from a PR-interval GWAS [30]. These SNPs are a distance of 62,252 bp from each other, located on Chromosome 5 at positions 173216115 and 173153863, respectively, and have an *R*^2^ of 0.19 in Hapmap samples with European ancestry [65]. The SNP rs255292 is located within the gene *BNIP1* and is 9,421 bp its transcription start site of *BNIP1*. The SNP rs4868243 lies between *BNIP5* and *NKX2–5* and is 71,673 bp and 15,994 bp from the transcription start sites of *BNIP1* and *NKX2–5*, respectively. This locus contains 6 protein-coding genes, of which *BNIP1* is the closest to a GWAS SNP.

The gene selected by SigNet at this locus is *NKX2–5*. It forms protein-protein interactions with HAND1 and ID2, selected at other information-poor loci, and is a transcriptional regulator of *TBX5*. The NKX2–5 protein is a homeobox transcription factor whose mutations affect cardiac development [66]. The regulated gene *TBX5* also encodes a transcription factor that itself regulates cardiac development [67]. Thus, evidence for *NKX2–5* as the causal gene is strong. Nevertheless, BNIP1 has a physical interaction with SCN3B, which has Mendelian evidence, and this locus may contain multiple causal genes.

## Discussion

The GWAS era has provided statistically reproducible associations between genetic variants and human biomedical phenotypes, including disease and disease risk. Determining how these variants have their effects is a basic step towards using these findings to improve basic understanding and advance human health. The SigNet method connects variants to likely causal genes with a Bayesian framework that integrates GWAS summary data with gene regulatory interactions and protein-protein interactions, selecting the most likely gene at each locus in the context of genes selected at other loci. It augments methods that focus primarily on within-locus information, such as Mendelian evidence, protein functional effects from variants that change amino acid sequence, colocalization, and chromatin state. By using information from evidence-rich loci to bias gene selection at evidence-poor loci, the method selects genes that differ from a common default approach of selecting the closest gene. Pathway enrichment analysis indicates that the results provided by SigNet are higher quality, and the literature review provides evidence for improved selection of causal genes. Our method, which learns from genes that have strong functional evidence, is complementary to data-driven approaches such as a recent polygenic priority score method PoPS [23]. We find that SigNet performs better than PoPS at loci where functional information provides strong evidence for a particular candidate. The network used by SigNet to generate a score may also help in inferring mechanistic interactions between genes and proteins contributing to a GWAS phenotype.

Improvements could include replacing binary features with real-valued features. While we set a genome-wide significance threshold on GWAS SNPs to include, we do not include quantitative information about the chi-square value or estimated regression coefficient. These could be included and could improve results for loci with multiple SNPs. Similarly, we could incorporate the score calculated by colocalization methods.

This method could be extended to include other sources of information. A property related to distance is co-occurrence of a SNP and a gene in a topologically associating domains (TADs). Flanking regions could be defined by TADs rather than by a fixed distance cutoff, although even within a TAD we might still anticipate an overall bias for causal genes to be closer to a SNP. Examining genes within the same TAD as a SNP has been helpful in identifying candidate genes [68–70]. Functional studies have shown that transcription factor binding sites are often within the same TAD as the regulated gene [71, 72]. One complication of incorporating TADs is that chromatin structure depends on the tissue or cell type and development stage. The tissue, cell, or developmental stage relevant to a particular association may not be known. An effective approach could be to learn the cell and tissue type along with building a model for the active SNPs. Learning the cell type could also help identify the best data sets to use for colocalization. Similarly, tissue-specific versions interaction databases could be incorporated [36], and single-cell data may be an additional source of information.

Of course, if TAD-related predictions are provided by other methods, these predictions could be readily incorporated along with colocalization in our naïve Bayes framework. Similarly, existing methods that aggregate within-locus information to provide a single summary score could be incorporated. The naïve Bayes approach assumes statistical independence between different evidence types. Aggregating methods would require greater attention to non-independence. One approach could be to model joint distributions of features drawn from the same data, for example a joint distribution for SNP-level and TWAS-level methods for colocalization, or to generalize from naïve Bayes to a more general functional form that accounts for non-independence. Deep learning could be considered, but the data available may not yet be sufficient for the bias-variance tradeoff.

A more fundamental improvement would be to lift the restriction of exactly one gene selected at each locus. Several of the loci in this study contain multiple genes with strong evidence for causality, including multiple genes with Mendelian evidence. Our method already provides scores and probabilities for all genes within a locus, but only allows one to be active at a time. An approach could be to include the number of active genes at a locus as a variable to be optimized, with a meta-parameter describing the distribution of active genes per loci. We have developed similar methods to estimate the number of independent effects at GWAS loci [73, 74].

Finally, our search for causal genes was limited to protein-coding genes. We did not include structural RNA genes, anti-sense RNAs, and long non-coding RNAs. Other RNA genes could be intriguing to include, particularly with appropriate interaction data for cis-regulation within a locus or trans-interactions across loci. Within a locus, anti-sense regulators could be connected with their cognate protein-coding genes. Regulatory RNAs could be connected to their targets at other loci, similar to gene-regulatory interactions of transcription factors. Known physical interactions between long non-coding RNAs or other RNA species and their protein binding partners could be included alongside protein-protein physical interactions.

## Conclusion

The SigNet method connects GWAS variants with the most likely causal gene at each GWAS locus, using genes selected at information-rich loci to bias the selection of genes at information-poor loci. The method improves on pathway enrichment obtained using a default approach of selecting the gene closest to a GWAS SNP and augments methods that use colocalization and other within-locus information. Applications to cardiovascular phenotypes provide new evidence for causal genes. Our results also highlight several GWAS loci that may include multiple causal genes. Methods that can learn the number of causal genes within each GWAS locus could be the most important next step in causal gene prioritization.

## Supporting information

S1 Table Summary table. Summary table of loci, genes, functional evidence, and SigNet gene selection for 100 independent runs.
(TXT)

S2 Table Table columns. Definitions of column headers in the summary table.
(TXT)

S3 Table Comparison table. Summary table joining SigNet and PoPS results.
(TXT)
